# Association between napping and type 2 diabetes mellitus

**DOI:** 10.3389/fendo.2024.1294638

**Published:** 2024-03-25

**Authors:** Hongyi Liu, Yingxin Wu, Hui Zhu, Penghao Wang, Tao Chen, Anyu Xia, Zhijia Zhao, Da He, Xiang Chen, Jin Xu, Lindan Ji

**Affiliations:** ^1^ School of Public Health, Health Science Center, Ningbo University, Ningbo, Zhejiang, China; ^2^ Department of Internal Medicine, Health Science Center, Ningbo University, Ningbo, Zhejiang, China; ^3^ Department of Clinical Medicine, Health Science Center, Ningbo University, Ningbo, Zhejiang, China; ^4^ Department of Obstetrics and Gynecology, Yinzhou District Maternal and Child Health Care Institute, Ningbo, Zhejiang, China; ^5^ Zhejiang Key Laboratory of Pathophysiology, Health Science Center, Ningbo University, Ningbo, China; ^6^ Department of Biochemistry and Molecular Biology, School of Basic Medical Sciences, Health Science Center, Ningbo University, Ningbo, Zhejiang, China

**Keywords:** napping, nighttime sleep duration, sleep pattern, type 2 diabetes mellitus, interaction

## Abstract

As the incidence of type 2 diabetes mellitus (T2DM) is increasing rapidly and its consequences are severe, effective intervention and prevention, including sleep-related interventions, are urgently needed. As a component of sleep architecture, naps, alone or in combination with nocturnal sleep, may influence the onset and progression of T2DM. Overall, napping is associated with an increased risk of T2DM in women, especially in postmenopausal White women. Our study showed that napping >30 minutes (min) increased the risk of T2DM by 8-21%. In addition, non-optimal nighttime sleep increases T2DM risk, and this effect combines with the effect of napping. For nondiabetic patients, napping >30 min could increase the risks of high HbA1c levels and impaired fasting glucose (IFG), which would increase the risk of developing T2DM later on. For diabetic patients, prolonged napping may further impair glycemic control and increase the risk of developing diabetic complications (e.g., diabetic nephropathy) in the distant future. The following three mechanisms are suggested as interpretations for the association between napping and T2DM. First, napping >30 min increases the levels of important inflammatory factors, including interleukin 6 and C-reactive protein, elevating the risks of inflammation, associated adiposity and T2DM. Second, the interaction between postmenopausal hormonal changes and napping further increases insulin resistance. Third, prolonged napping may also affect melatonin secretion by interfering with nighttime sleep, leading to circadian rhythm disruption and further increasing the risk of T2DM. This review summarizes the existing evidence on the effect of napping on T2DM and provides detailed information for future T2DM intervention and prevention strategies that address napping.

## 1 Introduction

Type 2 diabetes mellitus (T2DM) is the most common form of diabetes and accounts for more than 90% of diabetes cases worldwide (1). During the past few decades, the incidence and prevalence of T2DM have risen alarmingly and become an ongoing global public health issue. According to the 10th edition of the International Diabetes Federation (IDF) Diabetes Atlas, 537 million adults aged 20-79 years had diabetes worldwide in 2021, and this number is predicted to rise to 783 million by 2045 (2). When poorly controlled, T2DM can lead to many serious complications, including diabetic nephropathy, diabetic peripheral neuropathy, and diabetic retinopathy (3). Therefore, there is an urgent need to propose effective treatment and prevention strategies for T2DM and its complications.

Population-based studies have reported a U-shaped association between nighttime sleep duration and T2DM (4, 5). Compared with a sleep duration of 7-8 hours (h), both short sleep duration (<5-6 h) and long sleep duration (>8-9 h) increased the risk of impaired fasting glucose (IFG) and the incidence of T2DM. In addition, the number of night shifts worked per month appeared to have a dose-dependent relationship with the risk of T2DM (6). Interestingly, an association between chronotype and T2DM risk was also observed in shift workers (7). That is, the risk of T2DM was further increased by a long history of shift work among people with morning chronotypes, whereas people with evening chronotypes had a lower risk (7). Furthermore, participants with poor sleep quality had a higher risk of T2DM than those with good sleep quality (8–10). Thus, current evidence suggests that sleep is strongly associated with the incidence of T2DM. However, because other aspects of sleep, such as napping, have seldom been studied, research has not yielded a full, integrative understanding of how sleep affects T2DM.

As a key component of sleep architecture, napping act independently or in conjunction with nocturnal sleep to affect T2DM. Emerging evidence suggests that extended napping may increase the risk of T2DM (11, 12). Previous meta-analyses investigated the association between napping and T2DM, but these studies primarily focused on simplistic dichotomous classifications of either napping or no napping, or nap duration ≥1 hour/<1 hour (13–15). A recent meta-analysis examining specific nap durations (<30 minutes, 30-60 minutes, >60 minutes) revealed that naps exceeding 30 minutes were significantly associated with an increased risk of developing T2DM (16). However, these studies failed to provide a robust argument for the influence of nap duration on T2DM risk. In addition, none of these systematic investigations have explored potential variations in the relationship between napping and T2DM based on ethnicity or menopausal status among women, nor have they investigated the combined impact of napping and nighttime sleep on T2DM. Therefore, this review conducted an extensive search across PubMed, Embase, and Web of Science databases to identify relevant studies concerning the association between napping and T2DM. The impact of napping on glycemic traits as well as its role in T2DM development and complications was thoroughly investigated while carefully analyzing potential influencing factors. These findings may contribute to elucidating the comprehensive effect of napping on T2DM.

## 2 Basic concepts of napping

### 2.1 Definition, types, and duration of napping

Napping typically refers to a brief period of sleep in the early afternoon, ranging from a few minutes to several hours in duration. There is currently no standard classification system for napping. In previous studies, napping habits have been classified differently because of the different purposes of the studies. Broadly, some studies divide napping behavior qualitatively into 2 groups (napping and no napping or habitual napping and occasional napping) (12, 17, 18), while others use quantitative strategies to investigate the frequency of napping (19–21) or directly inquire about the duration of naps. For the latter, napping types may be divided into 3 groups [0, ≤1-30 and >30 minutes (min) (22); 0, ≤1 and >1 h (11, 18, 23–25)], 4 groups [0, 1-30, 31-90 and >90 min (26); 0, 1-30, 31-60, and >60 min (27, 28); 5-30, 31-60, 61-90 and >90 min (29)], or even 5 groups [0, 1-30, 31-60, 61-90 and >90 min (30–33)] using different cutoff time intervals. Notably, the definition of napping varies from that of excessive daytime sleepiness. The term “excessive daytime sleepiness” refers to an overwhelming sensation of somnolence, a persistent inclination for uninterrupted sleep or the incapacity to remain alert during daylight hours. This condition is evaluated using the Epworth Sleepiness, where a score of 10 or higher indicates excessive daytime sleepiness and may indicate the presence of a sleep disorder, medical issue, or other contributing factors (34). Conversely, Daytime napping is a purposeful and brief period of restorative slumber that typically provides revitalization and rejuvenation.

### 2.2 The relationship between napping and nighttime sleep

As humans are a diurnal species, nighttime sleep accounts for the majority of human sleep, while napping is used as a supplement. However, with changes in lifestyles and increasing shift work, the relationship between napping and nighttime sleep is becoming more complicated. It is commonly assumed that daytime naps, especially long naps in the late afternoon, may interfere with nighttime sleep (35, 36). However, there is conflicting information regarding their effect on health in interaction with other factors (37, 38). This is because the effect of napping may vary depending on the duration of night sleep. Naps, especially long naps, may be harmful for a variety of health conditions in people who sleep too long at night, while napping may have a protective effect for those who have inadequate night sleep. Concordantly, a study of self-reports from 1,166 community-dwelling participants aged 75-94 years (y) (83.4 ± 5.3) showed that regular napping significantly increased the risk of death in long sleepers (>9 h per night) but decreased mortality in short sleepers (<7 h per night) (39). Another study confirmed that if people who slept <5 h at night did not nap, they were often sleepier during the day (40). These observations indicate that proper napping is considered desirable to compensate for the negative health effects of sleep deprivation during the night. Although seldom studied, the quality of night sleep could also contribute to the interaction. Therefore, the effect of napping on health should not be evaluated only in isolation; instead, it would be more reasonable for further studies to consider the joint effect of napping and nocturnal sleep.

### 2.3 General effects of napping on people

**Figure 1:**
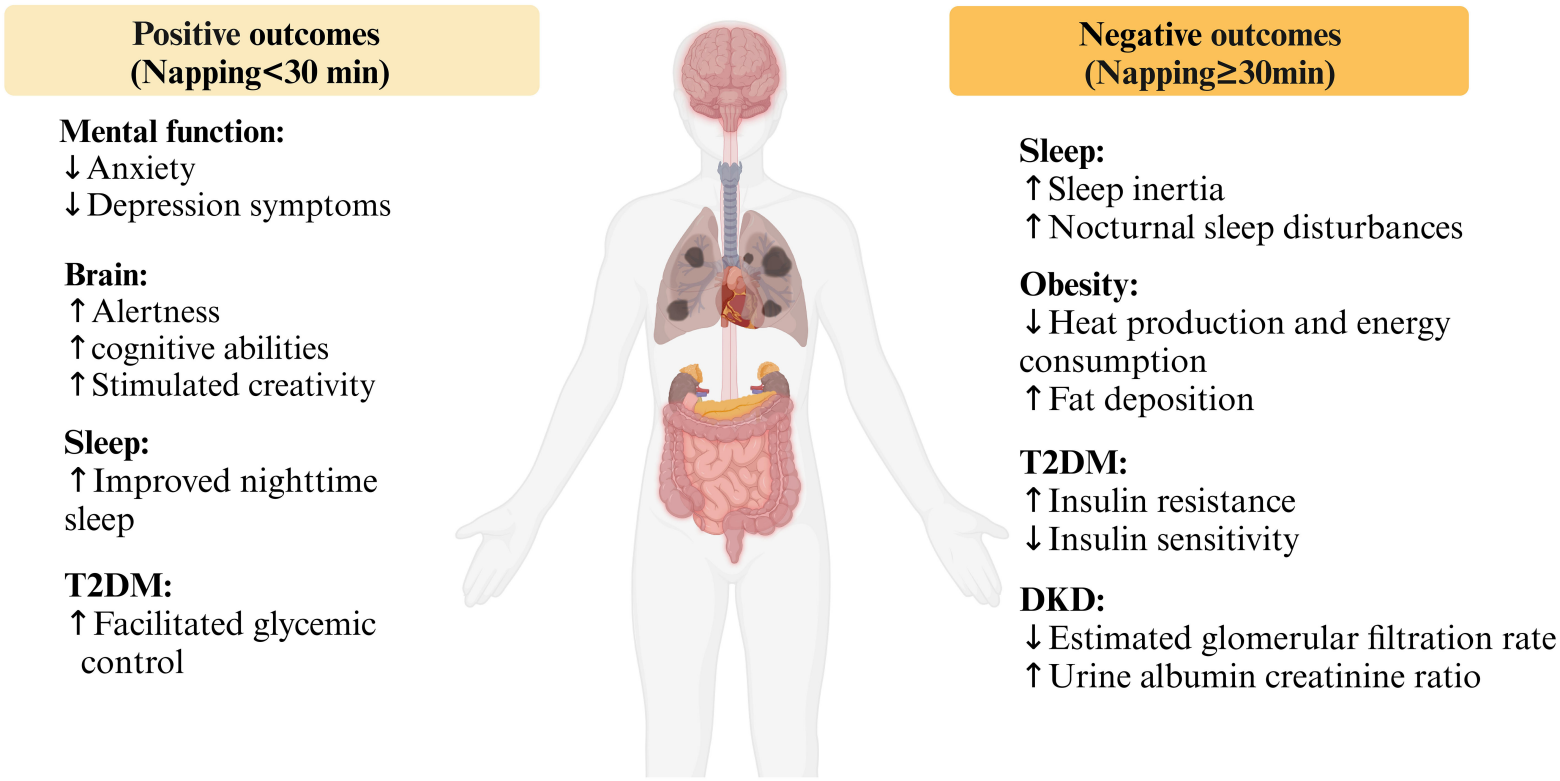
The positive and negative health outcomes of napping.

Napping poses both benefits and risks to health, and existing studies have reported inconsistent results due to different napping characteristics. Generally, the utilization of brief napping periods (<30 minutes) can contribute to the enhancement of alertness and concentration, thereby tasks (40–42). Moreover, various studies have demonstrated that taking a nap has been proven to enhance cognitive performance, facilitate the processing and storage of information, as well as improve learning outcomes and productivity (43–45). Napping can also alleviate feelings of fatigue and enhance mood states, thereby ameliorating symptoms of anxiety and depression, particularly in the midst of a demanding day or following inadequate sleep the previous night, invigorate you, diminish tension, and enhance your overall sense of well-being (46). The act of napping also serves to stimulate creativity and foster innovative thinking within the brain. Resting allows for brain recuperation and enhanced clarity ideas and effective problem-solving (44). On the contrary, late napping or longed naps can result in difficulties initiating sleep or experiencing shallow sleep during the night (47, 48). Additionally, they can foster indolence and procrastination, squandering valuable time and impeding the accomplishment of daily tasks and responsibilities. The overindulgence in napping may induce a phenomenon known as “sleep inertia” (44). The impact of sleep inertia can indeed be more severe than that of a night of sleep loss (49). The findings of a study indicate that brief naps lasting less than 15 minutes have the potential to mitigate sleep inertia (50). Overall, only proper napping duration can cause positive effects (**Figure 1**).

## 3 Factors influencing the association between napping and T2DM

### 3.1 Association between napping and T2DM in different races/ethnicities

Napping habits vary among different geographical locations, social circumstances, cultures, and populations. Daytime naps, especially shortly after lunch, are more commonly observed in countries with a napping culture, such as China. For them, midday naps are considered a healthy lifestyle practice, and most schools in China offer napping opportunities for children and adolescents (51). Statistically, approximately 20.3% of Chinese adults aged 30-79 y regularly take naps (52), and the percentage prevalence of habitual napping also increases with age (12, 23). In contrast, countries such as the US, UK, Germany and Finland do not practice habitual napping. Most European and American populations are more likely to nap unintentionally due to their current levels of exhaustion.

Racial differences in the relationship between nighttime sleep duration and T2DM do exist (53–55). Compared with White people, Black people have increased rates of short or long nighttime sleep durations and an accompanying increase in their risk of developing T2DM (56, 57). However, few studies focus on racial differences in the association between napping and T2DM. In a study of postmenopausal White, Filipina, and Black women aged 50-86 y (n = 1,658), daytime nap duration was associated with T2DM only in White women (22). After adjustment for covariates including nighttime sleep duration, White women who napped ≥30 min per day still had increased odds of developing T2DM (22). In contrast, the Sister Study, which included 39,071 eligible women from 2003 to 2009 (mean age 54.8 ± 8.8 y, with 87% self-identifying as White, 8% Black, and 5% Hispanic/Latina), showed that frequent napping (≥3 times/week) was associated with an overall 1.19-fold increase in the risk of T2DM (19). More specifically, for Black and Hispanic/Latina women, all sleep traits were significantly associated with T2DM risk after controlling for waist-to-hip ratio (WHR), body mass index (BMI) and other adiposity measures; but for White women, only napping frequency was significantly associated with T2DM risk (19). However, when within-race comparisons were made, only White women who napped frequently (≥3 times/week) had an increased T2DM risk (1.20-fold) (19).

### 3.2 Association between napping and T2DM in different ages and genders

**Figure 2:**
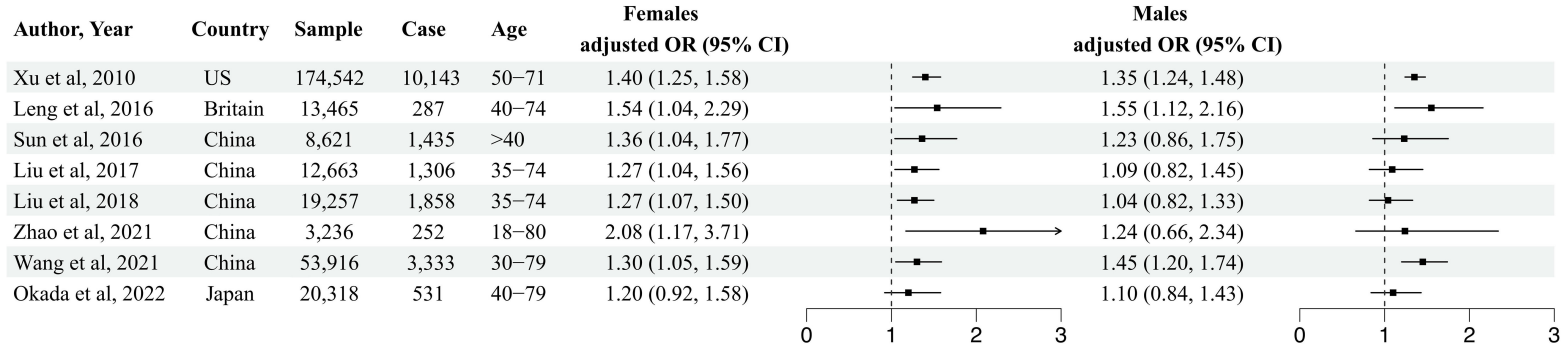
Effect of napping on T2DM by gender.

Factors such as age, gender, and female menopausal status may also contribute to inconsistencies in the various associations between napping and T2DM. Thus far, most cross-sectional studies have not observed a significant association between napping and T2DM in men (23, 24, 27, 32, 33). A cohort study including 53,916 Chinese participants (22,573 men, 31,343 women, mean baseline age was 52.0 ± 9.9 y) showed that male habitual nappers indeed had an increased risk of T2DM [OR (95% CI), 1.45 (1.20–1.74)] (12). The same phenomenon was also noted in the studies conducted by Leng et al. [1.55 (1.12, 2.16)] (17) and Xu et al. [1.35 (1.24, 1.25)] (18) (**Figure 2**). In contrast, most studies have observed significant associations between napping and an altered risk of T2DM in women. However, the results of a Japanese cohort study (N=20,318) indicated an insignificant correlation between napping and T2DM in women [1.20 (0.92, 1.58)] (58) (**Figure 2**).

**Figure 3:**
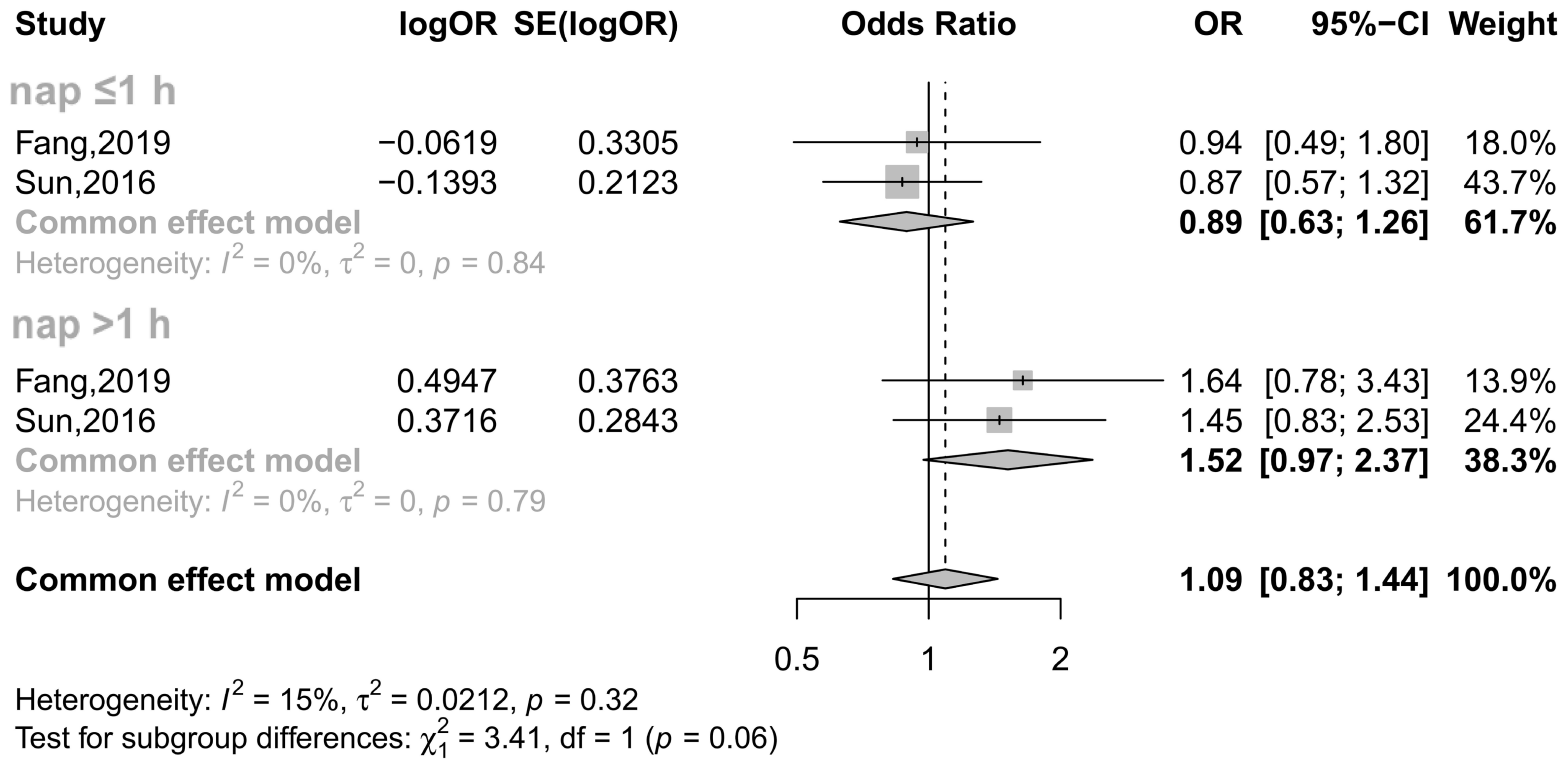
Effect of napping on T2DM incidence in premenopausal women. The plot shows the effect of different napping durations on the risk of T2DM in premenopausal women. CI, confidence interval; OR, odds ratio. The OR based on multiple regression analysis from the Fang study (24) and the adjusted OR from the Sun study (23) are reported.

Among female participants, the relationship again varies depending on menopausal status. A study including 6,178 eligible women (aged 40 y or older) showed that for premenopausal women, napping >1 h as opposed to not habitually napping at all was associated with an increased risk of T2DM in both unadjusted and exclusively age-adjusted models (23). However, after further adjustment for more confounders (BMI, smoking and drinking status, physical activity, nighttime sleep duration, education levels and retirement status), this statistical association disappeared (23). Further cross-sectional data from the China Health and Retirement Longitudinal Study showed that in the total sample of participants aged 45 years and older, a nap duration >60 min significantly increased the risk of T2DM (24). However, this statistical association disappeared in middle-aged premenopausal women (45-59 y) after stratification by age and menopausal status (24). For ascertainment, we carried out a meta-analysis of these two available studies (23, 24) and confirmed that there was no detectable association between napping and T2DM in premenopausal women (**Figure 3**).

Instead, studies have consistently concluded that postmenopausal women who habitually nap or those who nap for >1 h at a time are at increased risk for T2DM (**Supplementary Figure 2**) (23, 24). Fang et al. conducted an age-stratified analysis of 6,940 women aged over 45 y to assess the effect of napping on T2DM in postmenopausal women. Interestingly, their results showed that the association between napping >1 h and T2DM was present only in postmenopausal women aged 45-59 y (24). Other postmenopausal age ranges with different nap durations were also found to be specifically associated with T2DM risk, e.g., females aged ≥50 y who napped >1 h in Zhao et al.’s study [2.13 (1.17-3.88)] (27), women aged 45-54 y who napped ≥91 min in Liu et al.’s study (33), and postmenopausal women over 55 y of age who napped >1 h in Sun et al.’s study (23). Hence, the highest risk of T2DM was identified to occur with different nap durations at different postmenopausal ages in these studies. Among these studies, Fang et al. (24) and Sun et al. (23) clearly defined the postmenopausal age range, while other studies merely identified significant age ranges through stratification analyses. These results all imply that menopause indeed plays an important role in napping’s effect on T2DM risk. Next, Yan et al. carried out an in-depth study of this phenomenon and highlighted that the highest risk occurred in the initial postmenopausal stage (59). More specifically, premenopausal women have higher insulin sensitivity and a lower incidence of T2DM than age-matched men, and this advantage disappears after menopause, with an ensuing increase in impaired glucose homeostasis and the risk of T2DM (59). However, how the menopausal state affects the risks of T2DM through napping remains to be elucidated.

## 4 Effect of napping on blood glucose traits

### 4.1 Effect of napping on glucose metabolism in the nondiabetic population

It has been speculated that the high risks of elevated HbA1c levels and a high homeostasis model assessment of insulin resistance (HOMA-IR) index are results of inappropriate daytime napping (60). Thus, understanding how napping affects the glucose levels of nondiabetic people could help deepen the understanding of the effect of napping on the occurrence of T2DM.

As shown in **Table 1** (60–66), in general, longer napping duration or more frequent napping is related to increased HbA1c levels, hyperglycemia, IFG, impaired glucose regulation (IGR) and elevated IR risks. For example, consider HbA1c (63); a prospective study suggested that napping >30 min in the absence of sleep deprivation significantly increased the risk of abnormal glucose metabolism. In addition, for people with sleep deprivation, both naps >30 min and a lack of naps were associated with an increased risk of high HbA1c levels (>6.5%) over an average of 4.5 years of follow-up (63). In Chinese adolescents with adequate nighttime sleep, another study found that napping >3 times/week for >30 min/day may increase the risk of IFG (64). Here, nighttime sleep adequacy is inferred from total time in bed. Despite some bias, this metric still strongly suggests that for those adolescents with adequate nighttime sleep, prolonged (>30 min/day) or more frequent (>3 times/week) napping could significantly increase the risk of IFG (64). Overall, a detrimental effect of lengthy napping (>30 min/day) is strongly emphasized in these studies, regardless of adequate nighttime sleep or sleep deprivation. In contrast, it seems that napping for less than 30 min is likely to have a protective effect for both adequate and inadequate night sleepers. Nevertheless, as mentioned above, the correlation between napping and blood glucose characteristics needs to be considered in combination with nighttime sleep, since not taking naps was found to be associated with abnormal glucose metabolism for inadequate night sleepers only. Additionally, how napping interacts with night sleep to influence the blood glucose levels of nondiabetic individuals still needs further exploration.

**Table 1:** Effect of napping on blood glucose traits in non-diabetic population.

Author, year	Traits	Country/data source	Sample	Main results
Huang et al., 2014 (54)	HbA1c	China	A cross-sectional study involving 7,568 participants (59.6% females) aged 40-65 y in China.	Participants who reported napping had a higher risk of HbA1c >6.0%, IGR and IR.
Liu et al., 2022 (55)	HbA1c	UK Biobank	A mendelian randomization study based on UK Biobank (n = 336,999, mean age 57 y, 54.0% females).	Daytime napping (MVR 0.087 [0.081–0.093]; 1SMR main 0.090 [0.030–0.140]) was associated with higher HbA1c levels.
Zonoozi et al., 2017 (56)	HbA1c, fasting glucose and insulin	The British Regional Heart Study	A representative sample of British men aged 71-92 y with no history of heart attack or heart failure.	Napping duration >1 h showed significantly higher mean levels of HbA1c, fasting glucose and insulin.
Li et al., 2016 (57)	HbA1c, HOMA-IR index	China	5,845 Chinese (46.8% females; 30–65 y of age) based on the cohort study.	After an average of 4.5 y of follow-up, >30 min of daytime napping was significantly associated with an elevated HbA1c level (>6.5%) and high HOMA-IR index in overall.
Ji et al., 2019 (58)	IFG	China	The sample comprised 625 early adolescents (12.3 ± 0.6 y) with 45.3% (n = 283) girls from China.	Early adolescents who napped 3-4 days/week (OR = 1.72, *P* < 0.001), 5-7 days/week (OR = 1.34, *P* = 0.02) or ≥31 min/nap (OR = 1.52, 1.56, *P* < 0.05) were associated with increased likelihoods of IFG compared to non-nappers.
Al-Abri et al., 2022 (59)	HbA1c	Oman	A cross-sectional descriptive community-based study (n = 405, 48.0% female, mean age 32.8 ± 11.5 y) among the Arab population.	Napping >1 h is correlated with increased HbA1c in participants with normal night sleep duration in young and middle-aged adults.
Zheng et al., 2021 (60)	hyperglycaemia	China	A cross-sectional analysis of 172,901 adults aged ≥40 y living in China.	Long napping durations (≥1 h) were associated with hyperglycaemia (OR = 1.04, 95% CI = 1.01-1.09) compared with no napping without interactions from gender or age.

MVR, multivariable regression, 1SMR, one-sample Mendelian randomization, BMI, body mass index, IGR, impaired glucose regulation, IR, insulin resistance, IFG, impaired fasting glucose, TIB, time in bed.

### 4.2 Effect of napping on T2DM risk

Existing data show that longer napping duration (>30 min or >1 h), more frequent napping (>4 days/week), and habitual napping can all significantly increase the risk of T2DM (**Table 2**) (11, 12, 17, 18, 20–28, 30, 31, 37, 58, 67–73). To make it clearer, we conducted a meta-analysis that included 14 studies and 339,665 individuals (11, 18, 24–28, 30–33, 68, 69, 73). Because the classification of napping time is inconsistent among these studies, we first classified napping into <1 h and ≥1 h groups. The results showed that nap durations of both ≤1 h (OR = 1.16, 95% CI = 1.11-1.22) and >1 h (OR = 1.38, 95% CI = 1.23-1.55) increased the risk of T2DM (**Supplementary Figure 3**). This indicates that such a classification is too rough to provide useful guidance. Next, we classified nap duration using three cutoffs spaced at 30-min intervals, resulting the categories of ≤30 min, 31-60 min, 61-90 min, and ≥91 min. The results showed that there was no statistically significant association between napping ≤30 min and T2DM risk, while napping for 31-60 min (OR = 1.09, 95% CI = 1.02-1.16), 61-90 min (OR = 1.07, 95% CI = 1.00-1.15) or ≥91 min (OR = 1.20, 95% CI = 1.13-1.28) could significantly increase the risk of T2DM (**Supplementary Figure 4**). This result is consistent with the effect of lengthy napping (>30 min) on glucose metabolism in nondiabetic individuals. Thus, the different glycemic effects of napping ≤30 min and >30 min may account for part of the inconsistency. Future studies should use the vital classification time point of 30 min.

**Table 2:** Effect of napping on type 2 diabetes mellitus.

Author, year	Country/data source	Study design	Sample	Main results
Lin et al., 2021 (11)	China	Cohort study	2,620 elderly, 51.0% were males and the mean age was 66.9 ± 5.8 y.	Individuals with long daytime napping (>1 h/day) had increased risk of developing T2DM than non-nappers (adjusted RR = 1.52, 95%CI = 1.10, 2.10).
Wang et al., 2021 (12)	China	cohort study	The CKB study, 53,916 subjects (22,573 men and 31,343 women, 30-79 y).	5.11% of participants reported habitual daytime napping. Habitual daytime napping is positively associated with risk of T2DM in adults, except premenopausal females (OR = 1.39, 95%CI = 1.21, 1.59).
Leng et al., 2016 (17)	Britain	Cohort study	13,465 individuals (3,852 reported naps) from European Perspective Investigation into Cancer-Norfolk study, ages 40-74 y in UK.	Daytime napping was associated with an increased risk of T2DM (OR = 1.58, 95%CI = 1.23-2.03).
Xu et al., 2010 (18)	the U.S.	Cohort study	164,399 individuals at ages 50–71 y from the NIH-AAPP Diet and Health Cohort.	Longer daytime napping was associated with a higher risk for T2DM for those reporting <1 h (OR = 1.23, 95%CI = 1.18–1.29) and for those reporting ≥1 h (OR = 1.55, 95%CI = 1.45–1.66) of napping.
Lam et al., 2010 (20)	China	Cross-sectional study	Guangzhou Biobank cohort study, 19,567 participants (13,972 Chinese women and 5,595 men, 13,152 nap) ages ≥50 y.	Risk was increased for T2DM among older individuals who reported napping 4–6 d/week (OR = 1.36, 95%CI = 1.17–1.57) and daily napping (OR = 1.28, 95% CI = 1.15–1.44), and the ORs were 1.35 (95% CI 1.00–1.82) and 1.41 (1.11–1.81) for naps of ≤30 min and >30 min, respectively.
Hublin et al., 2016 (21)	Finland	Cohort study	The study population of the Finnish Twin Cohort in 1,990 included 12,244 subjects, ages 33-45 y in Finland.	The risk of T2DM was significantly increased only among those napping every or almost every day (OR = 1.86, 95% CI = 1.29–2.67).
Shadyab et al., 2015 (22)	southern California	Cross-sectional study	1,609 postmenopausal women (908 White, 330 Filipina, 371 Black) ages 50-86 y in United States.	The odds of T2DM were significantly higher among White women napping ≥30 min/day (OR = 1.74, 95% CI = 1.10-2.75) compared to White women without napping.
Sun et al., 2016 (23)	China	cross-sectional study	A community in Guang Zhou, 8,621 (2,443 men and 6,178 women, >40 y).	In multivariate logistic regression analysis, compared with no-habitual daytime napping postmenopausal women (n=3213), those with daytime napping more than 1 h (n=631) had higher prevalent T2DM (OR = 1.36, 95%CI = 1.04, 1.77). But no association was observed between men and T2DM (OR = 1.23, 95%CI = 0.86, 1.75).
Fang et al., 2019 (24)	China	cross-sectional study	The data from CHARLS, 6,940 participants (mean age 61.03 y).	Women who napped more than 60 minutes were more likely to report diagnosed T2DM. (OR = 1.39, 95%CI = 1.09, 1.76).
Ciren et al., 2021 (25)	China	cross-sectional study	CMEC study, 2,902 subjects (1146 men and 1756 women, aged 45-79 y).	Of all participants, 15.8% reported napping 1-50min, 12% reported napping ≥60min. Napping ≥60min was associated with T2DM (OR = 1.33, 95%CI = 1.01, 1.74).
Yin et al., 2018 (26)	China	cross-sectional study	The CHARLS study, 12,277 subjects (5,920 men and 6,357 women, mean age 59.2 y).	The weighted full adjusted ORs (95%CI) was 1.61 (1.22-2.13) for napping >90 min in diabetic patients.
Zhao et al., 2021 (27)	China	cross-sectional study	The data from four urban communities in Lanxi, Zhejiang Province, China. 3,236 participants (1,213 men and 2,023 women, 18-80 y).	Compared to the no daytime napping group (n=1272), people who napped during the daytime for >1 h (n=304) were independently associated with a greater prevalence of T2DM (OR = 1.59, 95%CI = 1.04, 2.43).
Fang et al., 2013 (30)	China	Cross-sectional study	Dongfeng–Tongji cohort, 27,009 retired employees (18,515 reported naps, mean age 63.6 y) in China.	Napping duration >30 min was significantly associated with the risk for T2DM (OR = 1.13, 95%CI = 1.02-1.25) and napping duration >60 min was significantly associated with the risk for IFG (OR = 1.11, 95% CI = 1.03-1.20).
Han et al., 2016 (31)	China	Cohort study	16,399 subjects (7,083 males and 9,316 females with a mean age of 62.5 y) from Dongfeng–Tongji cohort in China.	Long afternoon napping (>90 min) was associated with higher risk of incident diabetes (HR = 1.28, 95%CI = 1.03-1.59).
Picarsic et al., 2008 (37)	The LIFE-P study	Cross-sectional study	Community-dwelling older adults (n = 414, 225 reported naps), ages 70-89 y in United States.	Nappers were more likely to have T2DM (28% vs 14.3%, *P* = 0.007) than non-nappers.
Okada et al., 2022 (58)	Japan	cohort study	The Japan Collaborative Cohort Study, 20,318 participants (7,597 men and 12,721 women, 40-79 y).	No association was observed between napping and T2DM.
Kowall et al., 2016 (28)	Germany	cohort study	The Heinz Nixdorf Recall study, 4,814 participants (49.8% men, aged 45–75 years), with a median follow-up of 5.1 years.	Napping duration and frequency are not associated with an increased risk of T2DM.
Liu et al., 2018 (67)	China	cross-sectional study	The RuralDiab study, 19,257 subjects (7,007 men and 12,250 women, 35-74 y).	Napping duration of ≥91 min (n=4308) significantly increased the prevalence for T2DM (OR = 1.19, 95%CI = 1.04, 1.37).
Zhou et al., 2016 (68)	China	cross-sectional study	The Chinese Family Panel Studies, 13,469 participants (6,842 men and 6,627 women, >40 y).	Having over 60 minutes of daytime napping had weaker association compared with shorter duration of daytime napping (OR = 1.70, 95%CI = 1.12, 2.57). Daytime napping appears to be associated with elevated risk of incidence of T2DM (OR = 1.32, 95%CI = 0.80, 2.17).
Liu et al., 2022 (69)	China	cohort study	the REACTION cohort study, the remaining 11,539 subjects (4043 men, 7496 women), mean age 61 y.	The study did not find any significant association between napping and the risk of developing T2DM.
Zhou et al., 2023 (70)	China	cohort study	The UK Biobank, 435,342 subjects (194,677 men and 240,665 women, 40-69 y).	Higher daytime napping frequency (≥4 times per week) is associated with an increased T2DM risk (OR = 1.49, 95%CI = 1.41, 1.57).
Georgousopoulou et al., 2017 (71)	Global	cross-sectional study	The Mediterranean islands (MEDIS) study, 3,749 subjects (mean age >70 y).	No association between napping and T2DM was observed (OR = 1.11, 95%CI = 0.49, 2.54).
Stang et al., 2007 (72)	Germany	cohort study	the Heinz Nixdorf Recall Study, 4,472 subjects (2,125 men and 2,333 women, 45-74 y).	The risk of developing T2DM was significantly increased in both men and women who napped for more than 1 hour (men: 1.77 [1.57, 1.99]; women: 3.07 [2.18, 4.33]). However, in men, even napping for ≤1 hour also increased the risk (1.08 [1.03, 1.12]), whereas it acted as a protective factor in women (0.62 [0.53, 0.72]).
Ye et al., 2019 (73)	China	cohort study	A longitudinal (REACTION) study, 33,850 subjects (11,198 men and 22,652 women).	Napping is associated with a significantly elevated risk of developing T2DM, and this risk escalates over napping duration.

CMEC, China Multi-Ethnic Cohort; CHARLS, China Health and Retirement Longitudinal Study; RuralDiab, the Rural Diabetes, Obesity and Lifestyle study; CKB, China Kadoorie Biobank.

There are limited data on the combined effect of napping and nighttime sleep on T2DM. Generally, no napping and a nighttime sleep duration of 6-8 h is thought to be associated with the lowest risk of T2DM (11, 17). To be more specific, for people with enough night sleep, a longer napping duration (>1 h) will significantly increase the risk of T2DM, while for those with extremely long or short nighttime sleep durations (>8 h or ≤4 h), a napping duration >1 h will further aggravate this risk in comparison with the former individuals (**Table 3**) (11, 17, 18, 31, 58, 74). However, another study showed that poor quality and low quantity of nighttime sleep impair blood glucose control in T2DM, while napping can mitigate the deleterious effect of short nighttime sleep (<5 h) on glycemic control (75). Taken together, the extant data tend to support an adverse effect of longer napping duration (>1 h) for people with adequate or excessive night sleep. However, for people with inadequate night sleep, arguments still exist regarding whether napping could mitigate or worsen their risk of developing T2DM and how.

**Table 3:** The combined effect of napping and nighttime sleep duration on T2DM.

Author, year	Country	Study design	Sample	Main results
Lin et al., 2021 (11)	China	cohort study	2,620 eligible elderly (51.0% males, the mean age 66.9 ± 5.8 y) from the CHARLS, 18.4% napped >1 h.	Compared to non-nappers with 6–8 h of nighttime sleep, nappers who slept ≤4 h at night/day had significantly increased the risk of developing T2DM. Participants napped >1 h and slept over 6 h also showed higher T2DM risk.
Leng et al., 2016 (17)	British	cohort study	13,465 participants with complete information on daytime napping and the covariates from the European Perspective Investigation of Cancer-Norfolk (EPIC-Norfolk) cohort study.	The risk of developing T2DM more than doubled for those who took day naps and had less than 6 h of sleep, compared to those who did not nap and had 6-8 h of sleep.
Xu et al., 2010 (18)	the U.S.	cohort study	164,399 participants without diabetes and 10,143 participants with diabetic diagnosed after 2000 aged 50-71 y from the NIH-AARP Diet and Health cohort in United States.	Among participants with no napping, only short night sleeping was associated with higher occurrence of T2DM, whereas among those with ≥1 h of napping, both long and short sleeping was associated with higher risk, whereas individuals who napped ≥1 h during the day but slept <5 h at night had the highest risk (1.78-fold).
Han et al., 2016 (31)	China	cohort study	16,399 middle-aged and older Chinese (7083 males and 9,316 females with a mean age of 62.5 y) from the Dongfeng-Tongji cohort study.	Individuals with both napping >60min and sleep duration ≥10h had a 72% higher risk of incident diabetes than those with sleeping 7-8h and napping 0min (all above *P* < 0.05).
Okada et al., 2022 (58)	Japan	cohort study	20,318 participants (7,597 men, 12,721 women) aged 40–79 y from the JACC completed the 5-year follow-up survey and were included analyses.	Among the non-overweight, nappers who slept ≥10h had the highest risk of T2DM (OR = 2.84, 95%CI = 1.57-5.14), non-nappers who slept ≥10h (OR = 2.27, 95%CI = 1.27-4.06), and nappers who slept <10h (OR = 1.30, 95%CI = 1.03-1.64) was associated with T2DM.
Zhang et al., 2019 (74)	China	cross-sectional study	4,150 elderly Chinese (55.6% females), with an average age of 74 y living in China. 56.6% of the study subjects having habitual daytime napping.	Compared with the pattern of “daytime napping with short nighttime sleep”, people with” no daytime napping with long nighttime sleep (OR = 1.35, 95% CI = 1.01-1.80) or daytime napping with long nighttime sleep (OR = 1.36, 95% CI = 1.05-1.78)” had higher prevalence of T2DM.

To solve this problem, we conducted a meta-analysis of two studies (11, 31), which included 19,019 individuals (8,420 males and 10,599 females). Our results showed that regardless of how long nighttime sleep was, napping >1 h increased the risk of T2DM (**Supplementary Figure 5**), similar to the detrimental effect of lengthy napping (>30 min) on glucose metabolism in nondiabetic individuals. To be more specific, nighttime sleep duration <7 h and nap duration >1 h (OR = 1.71, 95% CI = 1.19-2.45), nighttime sleep duration 7-8 h and nap duration >1 h (OR=1.89, 95% CI=1.17-3.04), or nighttime sleep duration >8 h and nap duration >1 h (OR = 1.47, 95% CI = 1.17-1.85) increased the risk of T2DM. However, the heterogeneity was 65; the possible reason is that we combined the night sleep categories of ≤4 h and <7 h into a single category of <7 h for analysis, potentially contributing to the variability of the results.

Next, for further clarification, we examined the ≤4 h night sleep group in detail. In particular, a cohort study of older Chinese adults showed that participants who had a nighttime sleep duration ≤4 h and nap duration ≤1 h had a decreased risk of T2DM (0.92-fold), while those who slept for ≤4 h and napped for >1 h had a significantly increased risk (1.54-fold) (11). This suggests that for people who do not sleep sufficiently at night, proper napping (e.g., ≤1 h) works as a protective factor against T2DM, while excessive napping (e.g., >1 h) can increase this risk. Han et al. refined the <1 h nap duration interval to 30 min in a joint analysis of napping and nighttime sleep time. They found a protective effect of nighttime sleep duration <7 h and napping 1-30 min (OR 0.41, 95% CI 0.17-1.01). For nighttime sleep duration <7 h and napping duration 31-60 min, the OR nonsignificantly increased with increasing napping time (OR 0.74, 95% CI 0.45-1.24). Therefore, for those who do not sleep sufficiently at night, napping duration should be restricted to 30 min (31). However, this conclusion needs to be confirmed in a larger sample size in the future.

### 4.3 Effect of napping on glycemic control in T2DM patients

Aside from the effect of napping on all glucose metabolic traits and on T2DM risks, napping also has a deep association with glycemic control in T2DM patients (**Table 4**) (75–80). However, the current findings regarding the effect of napping on glycemic control in T2DM patients are inconsistent. Factors such as age, gender, nap duration, nighttime sleep duration, or the interaction of nap duration and nighttime sleep duration may contribute to the inconsistencies among these studies.

**Table 4:** Effect of napping on glycemic control in T2DM patients.

Author, year	Country/data source	Sample	Main results
Makino et al., 2018 (75)	Japan	457 Japanese patients, aged 29–90 y, diagnosed with diabetes or IGT.	Taking naps reduces the risk of short nighttime sleep for poor glycemic control (HbA1c > 7%), which indicates that midday naps in short nighttime sleepers with type 2 diabetes could be beneficial for their glycemic control.
Gozashti et al., 2016 (76)	Kerman	118 T2DM (90 males and 28 females, mean age 58 y).	A one-hour increment in sleep duration was associated with a 0.174% (1.4 mmol/mol) decrement in HbA1c. Moreover, participants who napped (66%) had a lower HbA1c (7.6 ± 1.0) compared to others (8.1 ± 1.3) (P=0.04).
Bawadi et al., 2021 (77)	Qatar	2,448 Qatari adults (1,000 men and 1,448 women) and long-term residents from 18–60 years of age with a history of T2DM.	The participants who consistently reported taking naps exhibited a significantly higher likelihood of experiencing poor glycaemic control (OR = 1.37, 95%CI = 1.05, 1.78).
Xue et al., 2022 (78)	UK biobank	12,997 T2DM (mean age >55 y).	Patients with T2D reporting regular daytime napping had higher odds of exhibiting an HbA1c value ≥7% compared with the never/rarely daytime napping group (OR = 1.17, 95%CI = 1.03, 1.32).
Kollannoor-Samuel et al., 2011 (79)	US	211 T2DM, mean age 56.4 y.	Napping for 30-60 minutes helps reduce the risk of high HbA1c levels (OR = 0.07, 95%CI = 0.01, 0.74).
Suárez-Torres et al., 2023 (80)	Mexico	202 T2DM (71% females, 20-60 y).	Napping significantly increased the risk of poor glycaemic control in T2DM patients (OR =2.9, 95%CI = 1.23, 6.76).

The criterion for poor glycaemic control was defined as having a HbA1c level of ≥ 7%.

Several studies have proposed a beneficial effect of napping on glycemic control in patients with T2DM. A cross-sectional study involving 118 patients (90 males and 28 females, mean age 58 y) with T2DM demonstrated that napping (with an average duration of 1.3 h) was associated with improved glycemic control (76). The same study also found that longer total sleep duration was linearly associated with better glycemic control and that napping may promote glucose metabolism by increasing total sleep duration (76). In this study, participants had an average nighttime sleep duration of 6.6 h. As mentioned above, the optimal nighttime sleep duration is 7-8 h; thus, this result suggests that napping may compensate for insufficient nighttime sleep for these patients and bring the total sleep duration into the optimal range, thus improving blood glucose control. Another study (398 people with T2DM and 43 people with abnormal glucose tolerance) also found that napping improved glycemic control (HbA1c >7%) in people with insufficient nighttime sleep (<5 h) (75). Thus, for T2DM patients, the glycemic effect of short nighttime sleep could be compensated by napping for the proper duration to achieve a more appropriate total sleep time and thus achieve better blood glucose control. However, the appropriate nap durations for different conditions of inadequate night sleep remain to be elucidated.

In contrast, another study involving 2,500 T2DM patients aged 18-60 y showed a 37% increased risk of poorly controlled HbA1c (≥7%) in patients who reported “always” napping compared to those who reported “never/rarely” napping (77). In addition, a study including 12,997 T2DM patients from the UK Biobank showed that patients who reported regular napping had a higher risk of HbA1c ≥7% while on insulin therapy than those who never or rarely napped during the same period (78). However, researchers have noted that this result could be confounded by multiple factors, including old age, long night sleep duration, high obstructive sleep apnea (OSA) incidence and low physical activity (81). Here, long night sleep duration probably played an important role in exerting different combined glycemic effects with napping. Of course, other intervening factors, particularly physical activity (82), should also be controlled in future studies of the effect of regular napping on T2DM control.

### 4.4 Effect of napping on T2DM complications

Current data on the association between daytime napping and diabetic complications are limited. Diabetes-associated kidney disease (DKD) is the most common comorbidity of T2DM, but the causes of its onset and progression are not clearly understood. DKD is one of the main causes of end-stage renal disease worldwide (83). Hypertension (84) and poor glycemic control (85) are identified as the two main risk factors for DKD, and both of these risk factors are independently related to daytime napping (21, 86). Since the association between napping and poor glycemic control has been introduced earlier, we focus only on its association with hypertension and DKD-related traits here.

In the Dongfeng Tongji cohort study, researchers conducted face-to-face interviews with 27,009 participants at baseline and studied the association between napping and hypertension risk. Generally, a longer nap duration (>30 min) was related to a higher blood pressure level and a higher risk of hypertension. After adjusting for confounding factors such as general sociological characteristics, BMI, family history and physical activity, this association still exists (86). Franke et al. investigated 733 participants (61% men, mean age 66 ± 9 y, mean diabetes duration 10 ± 8 y) (87) and found that the prevalence of DKD was significantly higher in patients with longer nap duration. More importantly, longer nap duration was significantly associated with an impaired estimated glomerular filtration rate [B (95% CI) = -0.05 (-0.09; 0.00), *P* = 0.044] and increased urine albumin creatinine ratio [B (95% CI) = 0.01 (0.01; 0.02), *P* < 0.001] (87). However, the pathological mechanism by which napping affects DKD patients and ultimately causes renal failure is still unclear. Since only the effect of napping on DKD was reported, future studies are still needed to explore the effect of napping on the onset and progression of other diabetic complications.

## 5 Possible mechanisms of the effect of napping on T2DM

**Figure 4:**
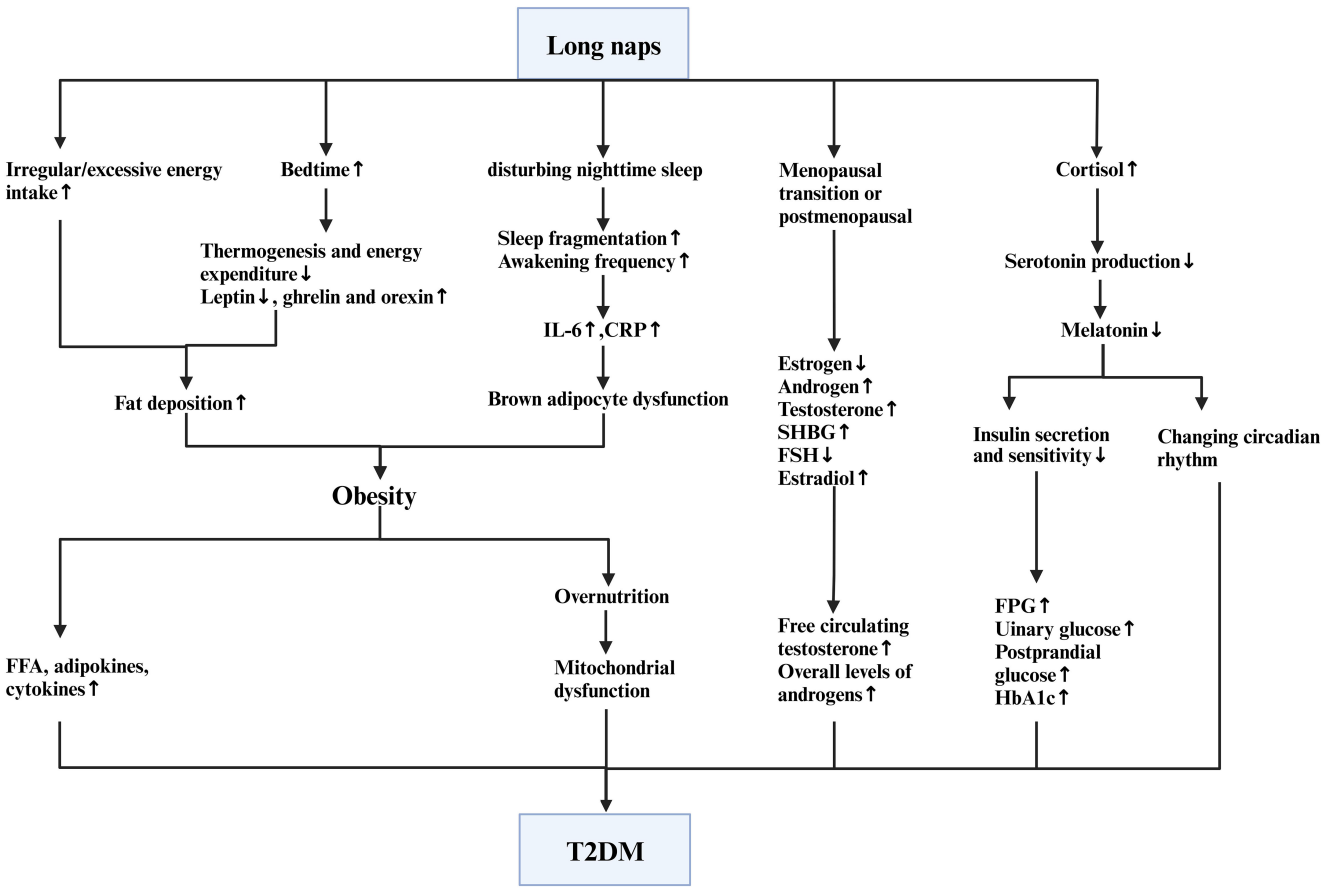
The influence of naps on T2DM.

Next, we elucidated some possible mechanisms through which napping exerts its effect on glycemic traits, T2DM and its complication risks (**Figure 4**). Additionally, we considered the potential synergistic effect of napping and nighttime sleep.

### 5.1 Link between increased obesity prevalence and T2DM

Evidence indicates that excessive nap duration adversely affects factors related to energy balance and weight control, thereby increasing the risk that obesity will develop (88). First, prolonged napping increases the amount of time spent stationary in bed, which directly leads to reduced thermogenesis and daily energy expenditure, lowered leptin levels, and elevated ghrelin and orexin levels that enhance fat deposition (81). Furthermore, extended daytime sleep may aggravate obesity by disturbing nighttime sleep (89). More specifically, lengthy naps may increase symptoms of insomnia at night, and fragmented sleep has been confirmed to be significantly related to increased BMI and obesity risk (90). Long naps may also enhance irregular and excessive energy intake and promote fat deposition (91). Taken together, the evidence indicates that longer nap duration has a strong effect on the occurrence of obesity.

The increased prevalence of T2DM is strongly associated with obesity. The global obesity epidemic largely explains the dramatic increase in the incidence and prevalence of T2DM over the past 20 years. Studies have shown that approximately 90% of T2DM cases are due to overweight (92). The link between obesity and T2DM involves two pathologies: insulin resistance (IR) and insulin deficiency (93). More specifically, obesity leads to persistently elevated plasma free fatty acid (FFA) levels and increased production of adipokines and cytokines, which could increase the risk of T2DM (94). Overnutrition leads to mitochondrial dysfunction, which in turn leads to IR and impaired β-cell function, contributing to the development of T2DM (95). Therefore, the metabolic disorders caused by obesity may lead to IR and progressive β-cell dysfunction. Reciprocally, reduced insulin function can increase the concentration of glucose, FFA and other nutrients, exacerbating overnutrition and thus creating a vicious cycle.

Physiologically, inflammation could partly explain the potential mechanistic link between naps and obesity. Esser et al. (96) proposed that higher levels of inflammatory biomarkers may play a role in the association between daytime napping and obesity. That is, longer naps may increase sleep fragmentation and the frequency of awakenings, leading to elevated interleukin 6 (IL-6) levels (97). IL-6 leads to an increased risk of inflammation, which may cause brown fat cells to dysfunction and render them unable to break down fat properly, resulting in the development of obesity (98). Additionally, excess daytime napping is reported to be associated with elevated C-reactive protein (CRP) levels among older people in the UK, particularly those who spend extreme amounts of time in bed at night (99). In a cohort of young people with a mean age of approximately 29 years, Mantua et al. found a linear increase in CRP with nap frequency (100). Elevated CRP levels could lead to an increased risk of obesity (101). Therefore, although the exact association of nap duration with IL-6 as well as CRP is unclear, excessive napping probably leads to increased levels of IL-6 and CRP, increasing the risk of obesity and subsequent T2DM.

### 5.2 Links between hormone dysregulation and T2DM

A wealth of studies have found a deep link between naps and T2DM in women, especially postmenopausal women. In the postmenopausal state, the declines in estrogen and progesterone as well as the symptoms of mood changes, fatigue, insomnia, anxiety, and depression that women experience during the transition phase of menopause predispose them to sleep disturbances and reduced insulin resistance regulation, thus increasing their risk of glucose metabolism disorders and T2DM (102–104). Although menopause (i.e., postmenopause) is clinically defined as the cessation of menstruation for at least 12 months, the menopausal transition itself actually begins 5-10 y earlier (105). Therefore, the association between prolonged napping and T2DM in postmenopausal women as opposed to premenopausal women should be attributed in part to abnormal hormone levels during the menopausal transition state.

During the menopausal transition, ovarian estrogen production decreases, testosterone levels increase, and sex hormone-binding globulin (SHBG) levels decrease (106, 107). After menopause, androgens increase, and the combination of increased testosterone and decreased SHBG leads to increased levels of free circulating testosterone and increased overall levels of androgens. These hormonal changes could further increase insulin resistance (108). It has also been suggested that women who reach menopause at <40 y have a significantly increased risk of T2DM compared to women who reach menopause in the typical age range of 45-54 y (109). Premature menopause is probably associated with more advanced hormonal changes as well as more abnormal sex hormone levels, leading to a higher prevalence of T2DM in postmenopausal women. These hormone changes are likely to worsen the adverse glycemic effect of sleep disturbance during this postmenopausal stage, thus helping to explain why napping >1 h significantly increases the risk of T2DM in postmenopausal middle-aged women. However, among postmenopausal women with high follicle-stimulating hormone (FSH) levels and low estradiol levels, the association between napping and T2DM seems more complicated. That is, postmenopausal individuals with lower FSH have higher FPG and HbA1c, as well as with higher rates of prediabetes and diabetes (110). In addition, the Women’s Health Study (111) and the Multi-Ethnic Study of Atherosclerosis demonstrate (112) that comparatively high estradiol levels are significantly associated with T2DM. Therefore, the integral effect of napping on glycemic traits among premenopausal, perimenopausal and postmenopausal women, together with its complex interaction with other intervening factors, should be further explored in the future.

In addition to the above sex hormone changes, cortisol may also play a role. The release of cortisol upon awakening in the morning, termed the cortisol awakening response (CAR), is followed by a decline throughout the day (113). A significant link between excessive daytime napping and elevated evening cortisol levels has been reported (113). A recent meta-analysis of napping and cortisol in children aged 0-5 y confirmed this connection and proposed the occurrence of the CAR after napping (114), supporting the causal relationship between them. Patients with the highest cortisol level have been found to have the highest fasting blood glucose, urinary glucose, and postprandial glucose as well as the highest HbA1c levels (115). Mechanistically, excessive cortisol can lead to increased risks of abdominal obesity and IR, which will ultimately lead to T2DM (116). Therefore, longer daytime napping duration may increase the risk of T2DM through increased cortisol levels as well.

### 5.3 Link between circadian rhythm disruption and T2DM

Extended naps may also disrupt circadian rhythms, increasing nocturnal awakening and shortening night sleep duration (117). This would lead to β-cell dysfunction, hyperglycemia, impaired glucose tolerance (118), increased HOMA-IR (119), and increased glycated hemoglobin levels, thus increasing the risk of T2DM (120).

Melatonin, as the sleep hormone, controls the circadian rhythm of the body and is secreted in humans primarily in the dark of night to support sleep (121). The master clock that controls circadian rhythms is located in the suprachiasmatic nucleus (SCN) of the hypothalamus. Prolonged napping increases cortisol, which then inhibits serotonin production, while insufficient serotonin results in decreased melatonin production (122). Reduced or delayed secretion of melatonin affects the SCN clock, thus changing the circadian rhythms in humans (123) and producing a profound effect on sleep patterns. Furthermore, melatonin can promote insulin secretion and sensitivity and reduce IR (124). When melatonin secretion is reduced, insulin secretion and sensitivity are weakened correspondingly, allowing elevated blood glucose and ultimately T2DM occurrence (125). Interestingly, it has been observed that melatonin levels decrease drastically in the first 15 years following menopause (126). This change causes circadian rhythm disturbances and sleep disorders during the menopausal stage and compounds the increase in T2DM risk among postmenopausal women (127).

The effect of napping on melatonin secretion has also been confirmed within young populations. A study involving 20 healthy children (11 females, aged 30-36 months) showed that toddlers who took naps had later bedtimes, later sleep onset times, longer sleep latency and shorter nighttime sleep, together with a later onset of melatonin, resulting in a delayed circadian rhythm (128). It has also been suggested that napping more often in a week may have a cumulative effect on delayed melatonin onset through an association with later bedtimes (128). In summary, napping may affect the onset of melatonin release as well as the quantity of melatonin production by affecting nighttime sleep, thus leading to circadian rhythm disturbances and further increasing the risk of T2DM.

## 6 Conclusion and summary

In conclusion, napping habits vary according to geographical location, social environment, culture and population characteristics. There are gender and ethnic differences in the association between napping and T2DM. In particular, the association between napping and T2DM is more pronounced in women than in men. When combinations of gender and ethnicity were considered, this risk was found to be particularly pronounced in postmenopausal White women. For napping alone, our study showed that napping ≤30 min did not increase the risk of T2DM, whereas napping >30 min increased the risk of T2DM by 8-21%. In addition, napping and nighttime sleep were observed to have a combined effect on glycemic control. That is, extreme nighttime sleep duration (<5-6 h or >8-9 h) itself increases the risk of T2DM, while napping >1 h exacerbates this risk. For nondiabetic individuals, napping duration may be an independent risk factor for poor glycemic control, and napping >30 min could increase the risk of high HbA1c levels and IFG, which would increase the risk of developing T2DM later on. In diabetic patients, prolonged napping may further impair glycemic control and increase the risk of developing diabetic complications (*e.g.*, diabetic nephropathy) in the distant future.

The association between napping duration and T2DM may be explained by obesity, hormone disturbance, menopausal status, and circadian rhythm disruption. First, naps >30 min increase nocturnal sleep fragmentation and the frequency of awakenings, leading to elevated IL-6 and CRP levels, which then increase the risks of inflammation and obesity. Together, they can lead to insulin resistance and a resultant increased risk of T2DM. Second, in the menopausal transition and postmenopausal states, decreased estrogen and sex hormone-binding globulin and increased androgen and testosterone levels jointly lead to elevated levels of free testosterone and total androgens in circulation. These sex hormone changes together with changes in the levels of other hormones, such as cortisol, further increase insulin resistance and the risk of T2DM. Third, prolonged napping may also affect the production of melatonin and the onset of its release, which would then influence nighttime sleep, leading to circadian rhythm disturbances and further increasing the risk of T2DM.

This paper systematically elucidates the relationship between napping and T2DM with extant data and provides important suggestions for glycemic control. First, the quantity and quality of nighttime sleep should be ensured. If nighttime sleep is sufficient and of good quality, then individuals should either refrain from napping or restrict the duration of their naps to 30 min. In contrast, if nighttime sleep is insufficient, it needs to be compensated by appropriate napping (≤30 min). Second, if the duration of night sleep is excessive, then napping is not recommended. Nevertheless, the current research has limitations, such as the inconsistent classification of napping in the studies we included, which resulted in a high level of heterogeneity between studies. Next, studies should be conducted to explore the detailed characteristics of napping more thoroughly, including the start and end times and the interval between napping and lunch, which are seldom considered. Additionally, quantitative and qualitative measurements of glycemic change, such as continuous glucose monitoring (CGM), which can trace glycemic changes earlier and more accurately than other methods, should be used in the future to better investigate the effect of napping on T2DM.

## Author contributions

HL: Data curation, Writing – original draft. YW: Writing – original draft, Data curation. HZ: Writing – original draft, Data curation. PW: Writing – original draft, Data curation. TC: Writing – original draft, Data curation. AX: Writing – original draft, Data curation. ZZ: Writing – original draft, Data curation. DH: Writing – original draft, Data curation. XC: Writing – original draft, Data curation. JX: Writing – review & editing. LJ: Writing – review & editing.
