# Corrigendum: Association between napping and type 2 diabetes mellitus

**DOI:** 10.3389/fendo.2024.1413519

**Published:** 2024-04-19

**Authors:** Hongyi Liu, Yingxin Wu, Hui Zhu, Penghao Wang, Tao Chen, Anyu Xia, Zhijia Zhao, Da He, Xiang Chen, Jin Xu, Lindan Ji

**Affiliations:** ^1^ School of Public Health, Health Science Center, Ningbo University, Ningbo, Zhejiang, China; ^2^ Department of Internal Medicine, Health Science Center, Ningbo University, Ningbo, Zhejiang, China; ^3^ Department of Clinical Medicine, Health Science Center, Ningbo University, Ningbo, Zhejiang, China; ^4^ Department of Obstetrics and Gynecology, Yinzhou District Maternal and Child Health Care Institute, Ningbo, Zhejiang, China; ^5^ Zhejiang Key Laboratory of Pathophysiology, Health Science Center, Ningbo University, Ningbo, China; ^6^ Department of Biochemistry and Molecular Biology, School of Basic Medical Sciences, Health Science Center, Ningbo University, Ningbo, Zhejiang, China

**Keywords:** napping, nighttime sleep duration, Sleep pattern, type 2 diabetes mellitus, interaction

In the published article, there was an error in the Funding statement. The funding statement for the Ningbo Clinical Medical Research Center for Ophthalmology was displayed as “2023-D3”. The funding statement for the Beijing Zhongwei Joint Funds of the Zhejiang Provincial Natural Science Foundation of China (LBY24H040001, LBY24H040002) was displayed as “Zhejiang Basic Public Welfare Research LBY24H040001 and LBY24H040002”. The correct Funding statement appears below.

“The author(s) declare financial support was received for the research, authorship, and/or publication of this article. This work was supported by the Ningbo Youth Science and Technology Innovation Leaders Project (2023QL057), Technology Innovation 2025 Major Project of Ningbo (2021Z054), Graduate Student Scientific Research and Innovation Project of Ningbo University (IF2023057), Ningbo Clinical Medical Research Center for Ophthalmology (2022L003), and Beijing Zhongwei Joint Funds of the Zhejiang Provincial Natural Science Foundation of China (LBY24H040001, LBY24H040002).”

In the published article, the reference for Liu et al., 2022 in Table 2 was incorrectly written as “[710”. It should be “Liu H, Chen G, Wen J, Wang A, Mu Y, Dou J, et al. Association between sleep duration and incidence of type 2 diabetes in China: the REACTION study. Chin Med J (Engl). (2022) 135:1242–8. doi: 10.1097/CM9.0000000000001835”.

In the published article, the reference for Leng et al., 2016 in Table 3 was incorrectly written as “Yamada T, Shojima N, Yamauchi T, Kadowaki T. J-curve relation between daytime nap duration and type 2 diabetes or metabolic syndrome: A dose-response meta-analysis. Sci Rep. (2016) 6:38075. doi: 10.1038/srep38075”. It should be “Leng Y, Cappuccio FP, Surtees PG, Luben R, Brayne C, Khaw KT. Daytime napping, sleep duration and increased 8-year risk of type 2 diabetes in a British population. Nutr Metab Cardiovasc Dis. (2016) 26:996–1003. doi: 10.1016/j.numecd.2016.06.006”.

In the published article, the reference for Xu et al., 2010 in Table 3 was incorrectly written as “Chen GC, Liu MM, Chen LH, Xu JY, Hidayat K, Li FR, et al. Daytime napping and risk of type 2 diabetes: a meta-analysis of prospective studies. Sleep Breath. (2018) 22:815–24. doi: 10.1007/s11325-017-1528-z”. It should be “Xu Q, Song Y, Hollenbeck A, Blair A, Schatzkin A, Chen H. Day napping and short night sleeping are associated with higher risk of diabetes in older adults. Diabetes Care. (2010) 33:78–83. doi: 10.2337/dc09-1143”.

In the published article, the reference for Han et al., 2016 in Table 3 was incorrectly written as “Zhao X, Cheng L, Zhu C, Cen S, Lin W, Zheng W, et al. A double-edged sword: the association of daytime napping duration and metabolism related diseases in a Chinese population. Eur J Clin Nutr. (2021) 75:291–8. doi: 10.1038/s41430-020-00777-2”. It should be “Han X, Liu B, Wang J, Pan A, Li Y, Hu H, et al. Long sleep duration and afternoon napping are associated with higher risk of incident diabetes in middle-aged and older Chinese: the Dongfeng-Tongji cohort study. Ann Med. (2016) 48:216–23. doi: 10.3109/07853890.2016.1155229”.

In the published article, the reference for Okada et al., 2022 in Table 3 was incorrectly written as “Zheng B, Lin LL, Yu CQ, Lyu J, Guo Y, Bian Z, et al. Distributions and associations between duration of sleep, daytime naps and insomnia symptoms among Chinese adults. Zhonghua Liu Xing Bing Xue Za Zhi. (2017) 38:452–6. doi: 10.3760/cma.j.issn.0254-6450.2017.04.008”. It should be “Okada R, Teramoto M, Muraki I, Tamakoshi A, Iso H. Sleep duration and daytime napping and risk of type 2 diabetes among Japanese men and women: the Japan collaborative cohort study for evaluation of cancer risk. J Epidemiol. (2023) 33:562–8. doi: 10.2188/jea.JE20220118”.

In the published article, the reference for Zhang et al., 2019 in Table 3 was incorrectly written as “Li X, Pang X, Zhang Q, Qu Q, Hou Z, Liu Z, et al. Long-term single and joint effects of excessive daytime napping on the HOMA-IR index and glycosylated hemoglobin: A prospective cohort study. Med (Baltimore). (2016) 95:e2734. doi: 10.1097/MD.0000000000002734”. It should be “Zhang S, Xie L, Yu H, Zhang W, Qian B. Association between nighttime-daytime sleep patterns and chronic diseases in Chinese elderly population: a community-based cross-sectional study. BMC Geriatr. (2019) 19:124. doi: 10.1186/s12877-019-1136-9”.

The authors apologize for these errors and state that this does not change the scientific conclusions of the article in any way. The original article has been updated.

