# Supplementary material for: Association between napping and type 2 diabetes mellitus
Source: Front Endocrinol (Lausanne). 2024 Mar 25;15:1294638. doi: 10.3389/fendo.2024.1294638 (PMC10999583; doi:10.3389/fendo.2024.1294638)
Supplement: Supplementary file 1 [file DataSheet_1.docx]

Supplementary Material

# Research design and methods

- 1. **Literature search**

We conducted a comprehensive literature search of the PubMed, Embase, and Web of Science databases to identify published studies investigating the association between napping and type 2 diabetes mellitus (T2DM). The search terms used are provided in Table S1, and we limited our search to English language publications. Articles were initially screened based on their titles and abstracts, and those meeting the inclusion criteria underwent full-text review. Figure S1 depicts the results of our literature search and study selection process.

- 1. **Study selection**

The studies included in this meta-analysis met the following criteria: (1) they employed an observational study design, (2) the exposure of interest was napping, (3) the outcome measured was T2DM, (4) the investigators reported adjusted odds ratio (OR), related risk (RR), or hazard ratio (HR) along with a 95% confidence interval (CI), and (5) they were available as full-text articles. We excluded: (1) animal studies, clinical trials, reviews, commentaries, letters, meta-analyses, and studies investigating other associations; (2) duplicated reports; and (3) studies that reported incomplete data or lacked relevant data availability. Two authors independently assessed study eligibility. Any discrepancies regarding eligibility were resolved through consensus with another author acting as a third-party.

- 1. **Data extraction and quality**

Data were independently extracted by H-YL and Y-XW, with discrepancies resolved through discussion and consensus with either HZ or P-HW. The following information was extracted from each study: authors, year of publication, study name, study type, study location, sample size, participant age, categories of napping duration, and OR/RR/HR (95%CI). Cross-sectional studies were assessed using the Agency for Healthcare Research and Quality (AHRQ), with scores categorized as low (1-3), medium (4-7), or high-quality (8-11). Cohort and case-control studies were evaluated using the Newcastle-Ottawa Scale (NOS), with scores indicating low quality (0-3), medium quality (4-6), or excellent quality (7-9).

**1.4 Data synthesis and analysis**

All statistical analyses were performed using R 4.2.2 software. This mini meta-analysis aimed to quantitatively evaluate the correlation between napping and T2DM. The extracted OR (95% CI) were combined using the inverse variance method. Forest plots were utilized to visually present both individual and overall effect sizes. Heterogeneity was assessed using the Q statistic, with *I^2^* and *P* values used for quantification purposes. A fixed-effects model was employed when *I^2^* ≤ 50% and *P* ≥ 0.01, while a random-effects model was chosen if *I^2^* > 50% and *P* < 0.01 criteria were met. Subgroup analysis techniques were applied to explore potential sources of heterogeneity. The Egger's regression test was utilized to detect the absence of significant bias. If the P-value is greater than 0.05, it can be inferred that there is no publication bias present. The trim and fill method was employed to address potential publication bias effectively. Sensitivity analyses were conducted to assess the robustness of the combined results.

# Supplementary Figures and Tables

## Supplementary Figures


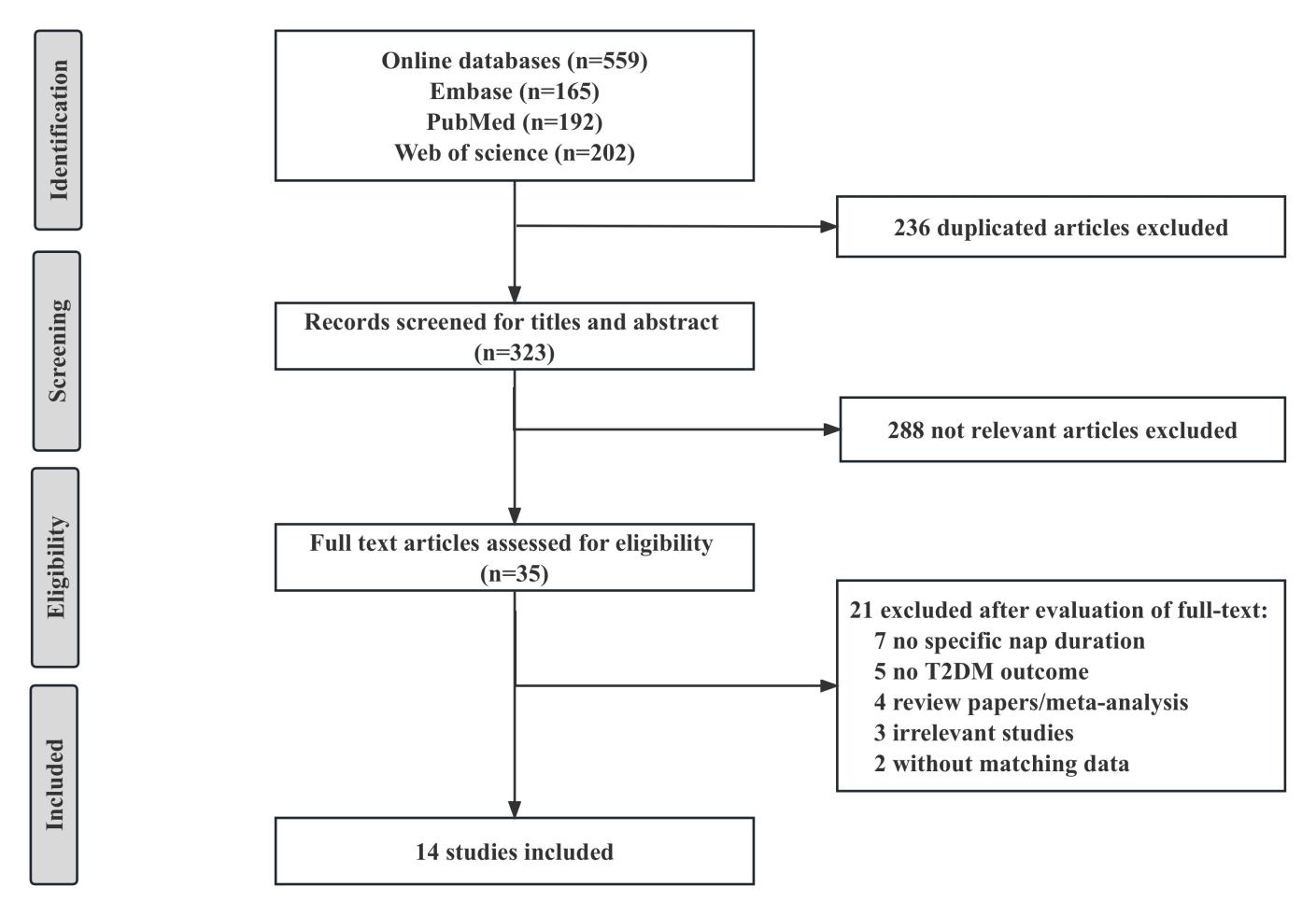


**Supplementary Figure 1.** Flow diagram of literature search and study selection.


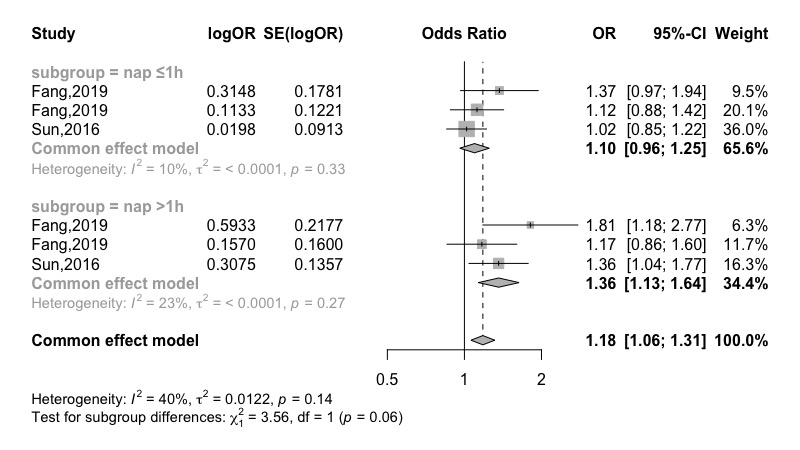


**Supplementary Figure 2.** Effect of napping on T2DM incidence in postmenopausal women. The plot shows the effect of different napping durations on the risk of T2DM in postmenopausal women. Postmenopausal women who habitually nap or nap for >1h are at increased risk for T2DM. Here, Middle-Aged Postmenopausal women and Older Postmenopausal women from the Fang study [1] were considered as postmenopausal women. CI = confidence interval. OR = odds ratio.


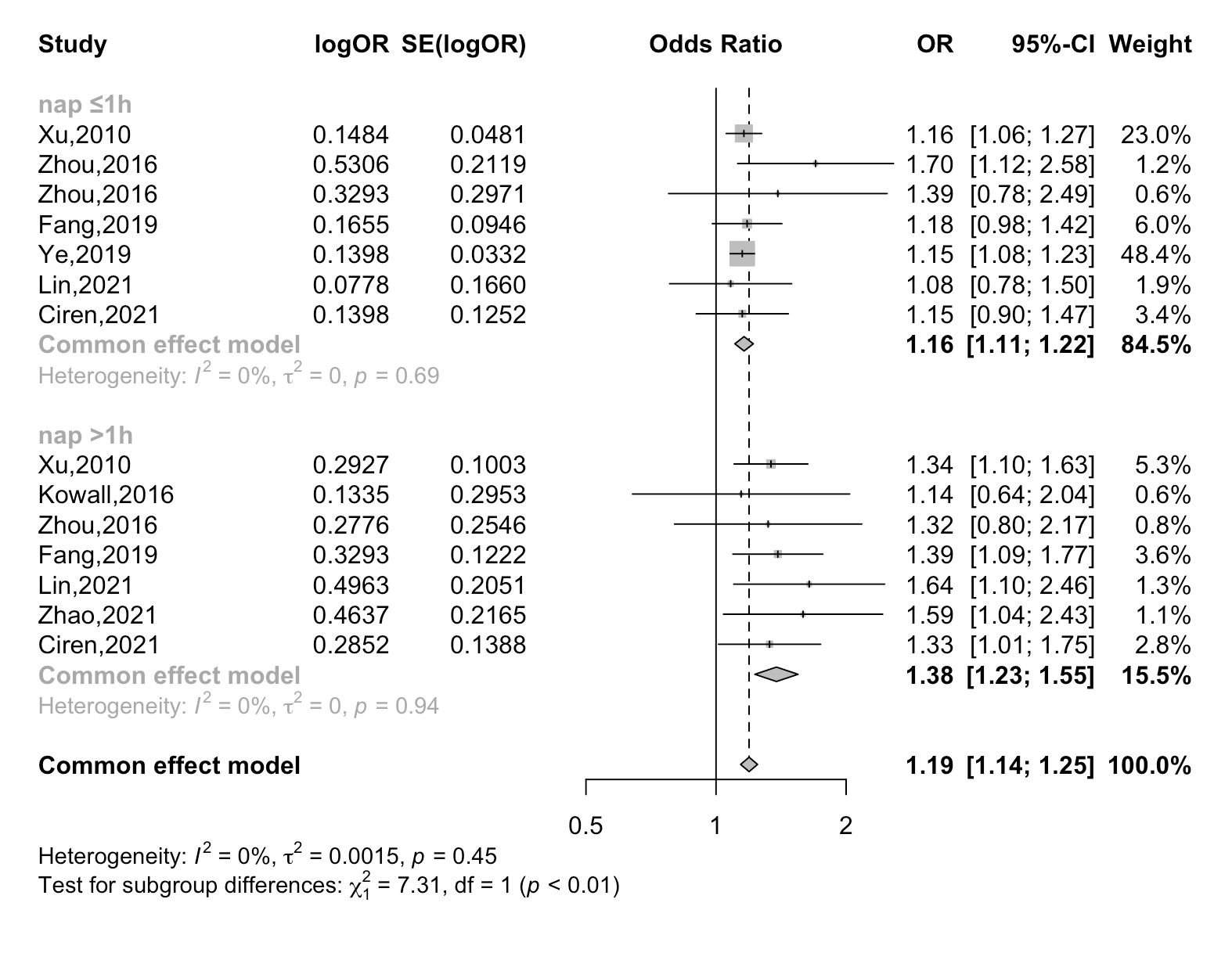


**Supplementary Figure 3.** The effect of napping duration on T2DM incidence (1h as time interval). The plot shows the risk of T2DM for participants with napping durations <1h and ≥1h. Both nap durations would increase the risk of T2DM.


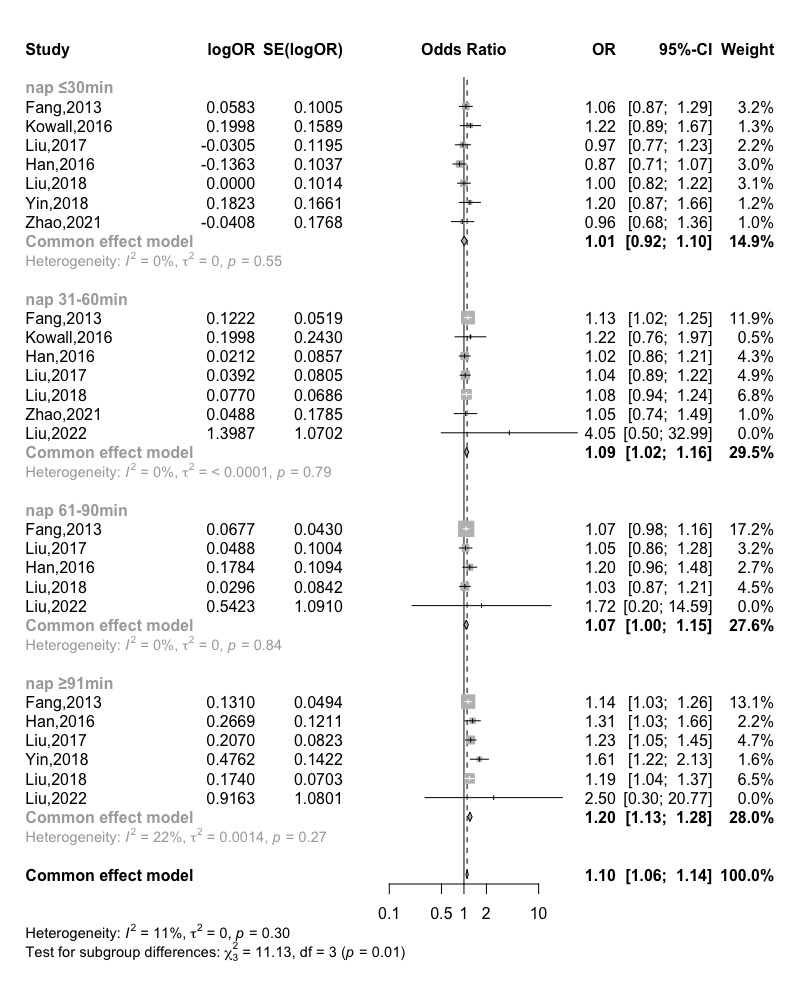


**Supplementary Figure 4.** The effect of napping duration on T2DM incidence (30min as time interval). The graph shows the risk of T2DM for participants who took a nap for ≤30min, 31-60min, 61-90min and ≥91min. Except of ≤30min, napping for 31-60min, 61-90min and ≥91min could significantly increase the risk of T2DM.


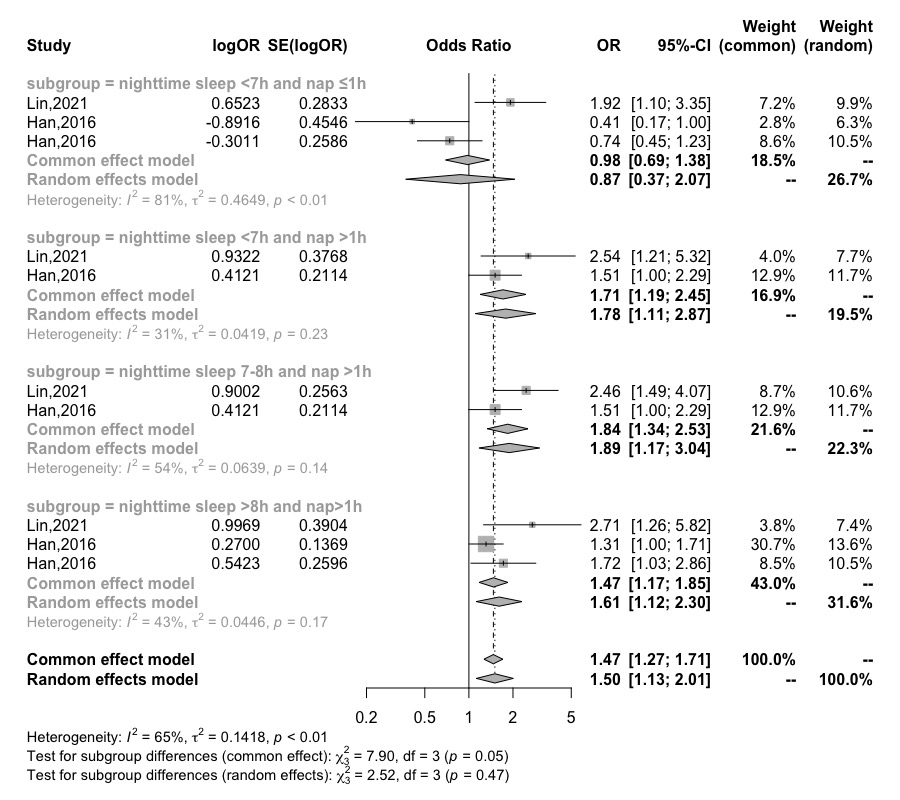


**Supplementary Figure 5.** The combined effect of nighttime sleep and napping on. The combined effect of different nighttime sleep durations and different nap durations on T2DM was derived from the limited data provided in the literature. The Han study [2] included “nighttime sleep <7h and nap 1-30min” and “nighttime sleep <7h and nap 31-60min”, which were considered as “nighttime sleep <7h and nap ≤1h”. “Nighttime sleep 8-10h and nap >1h” and “nighttime sleep ≥10h and nap >1h” were considered as “Nighttime sleep >8h and nap >1h”.


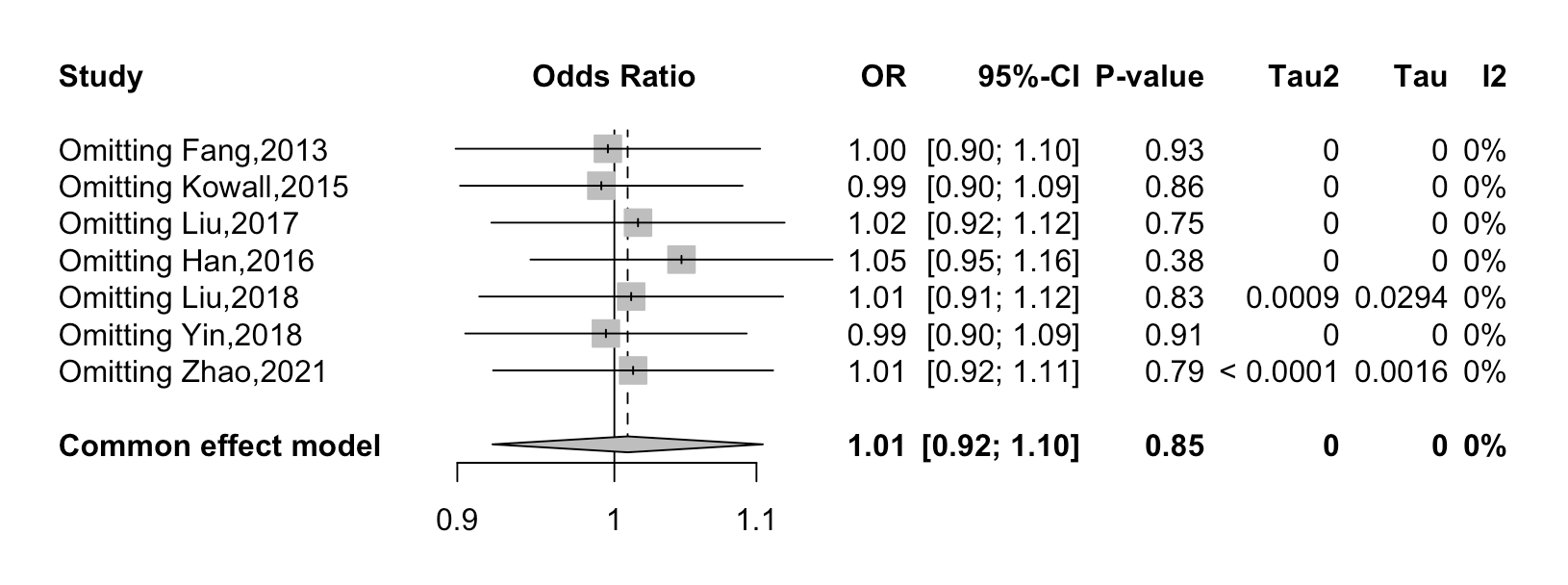
**Supplementary Figure 6.** Sensitivity analysis for the association between napping <30 min and risk of T2DM.
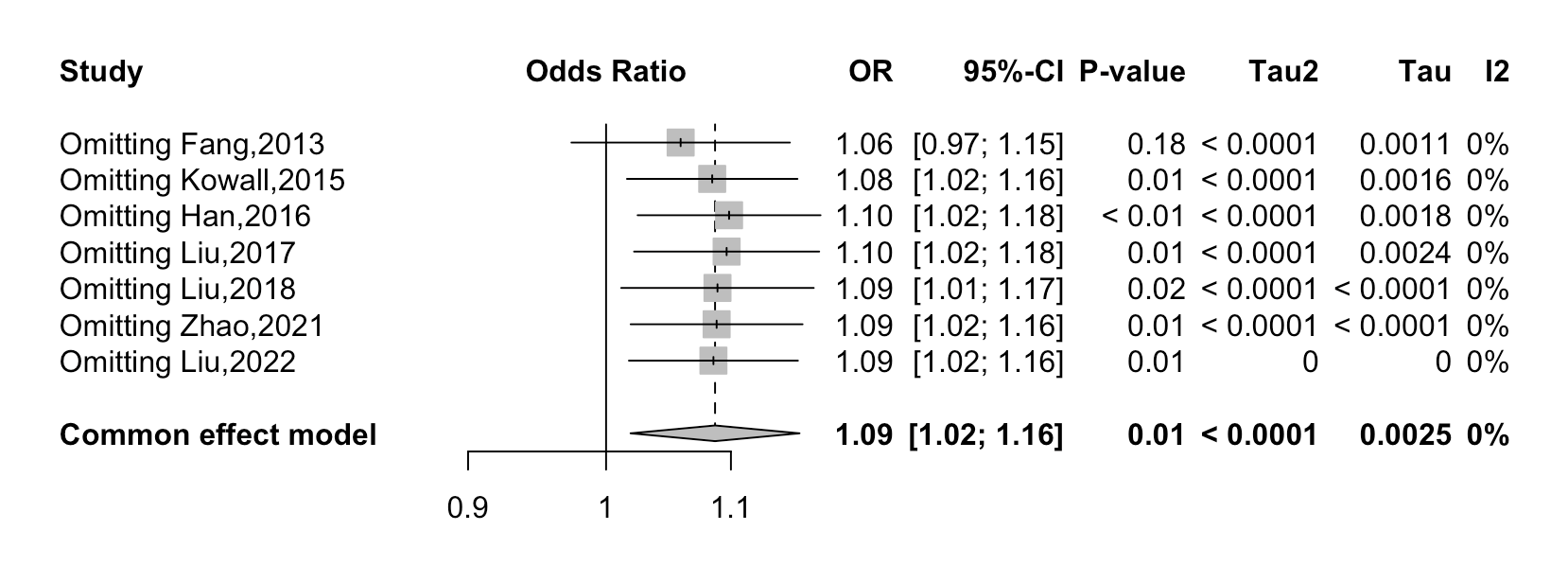


**Supplementary Figure 7.** Sensitivity analysis for the association between napping 30-60 min and risk of T2DM.


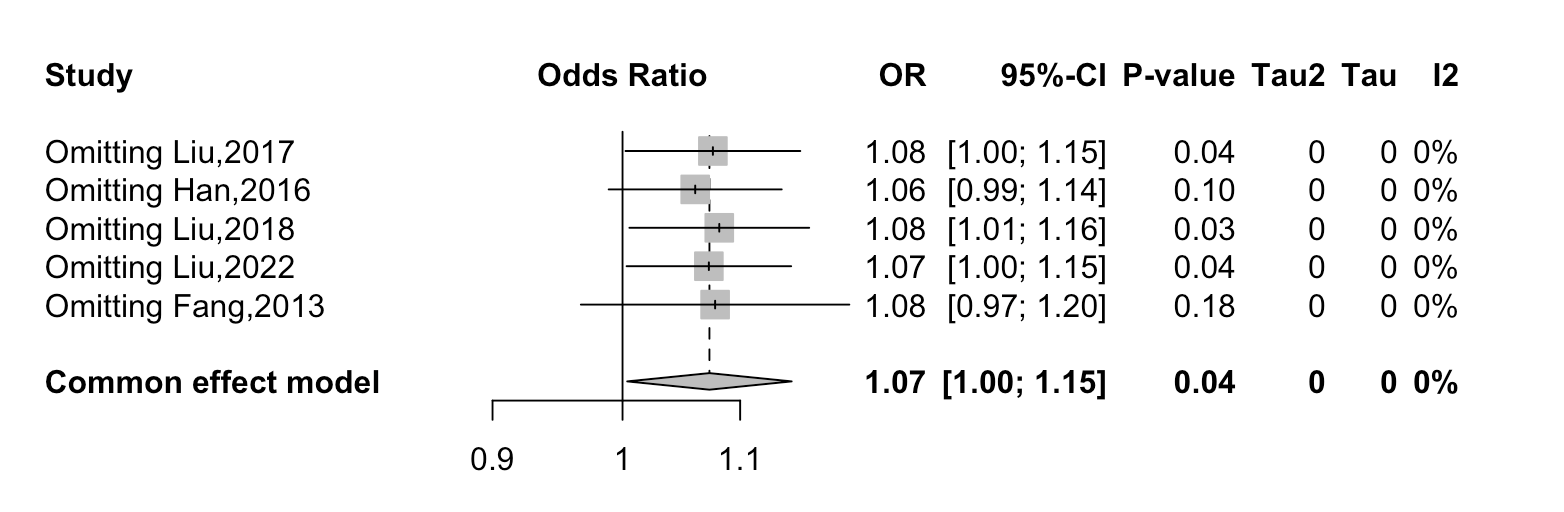


**Supplementary Figure 8.** Sensitivity analysis for the association between napping 60-90 min and risk of T2DM.
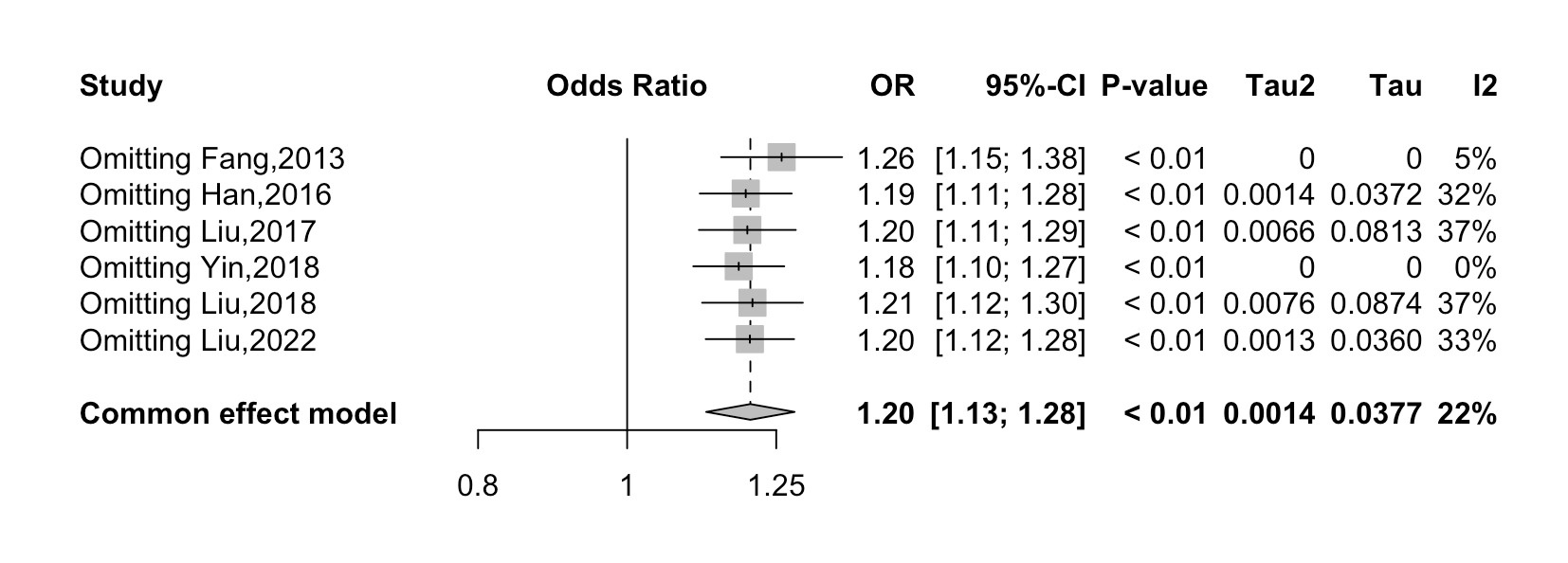


**Supplementary Figure 9.** Sensitivity analysis for the association between napping >90 min and risk of T2DM.


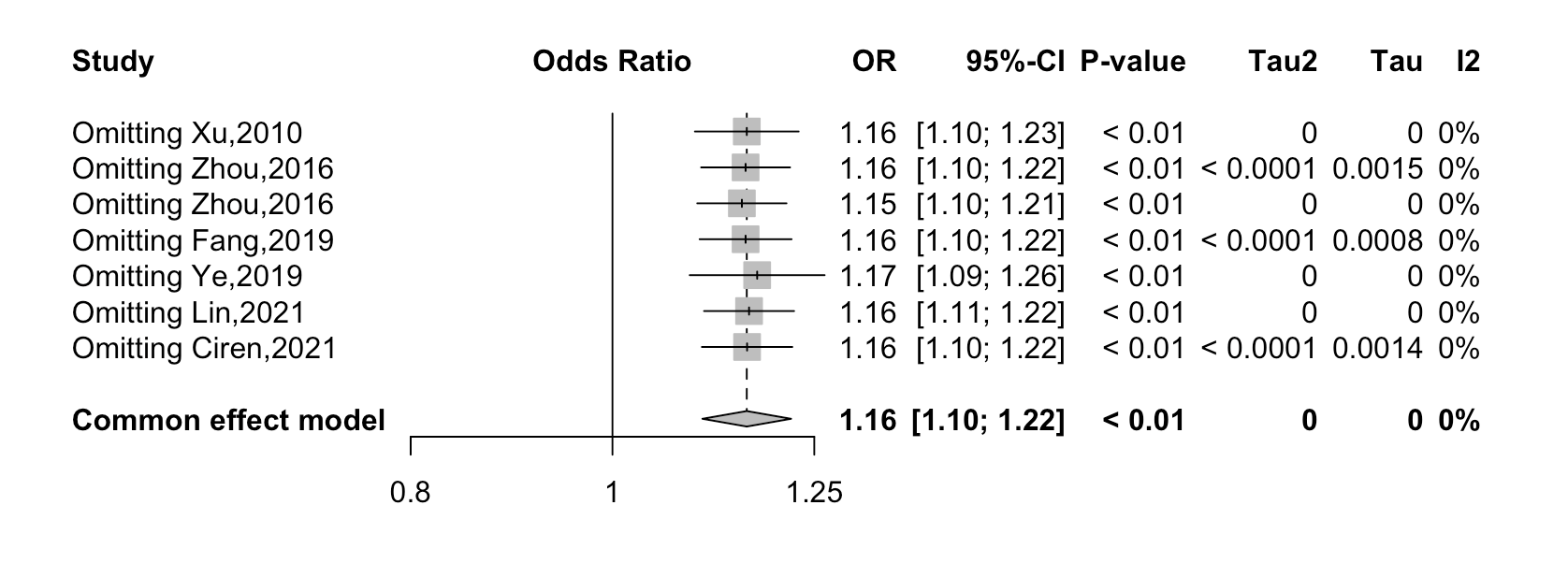


**Supplementary Figure 10.** Sensitivity analysis for the association between napping<1 h min and risk of T2DM.


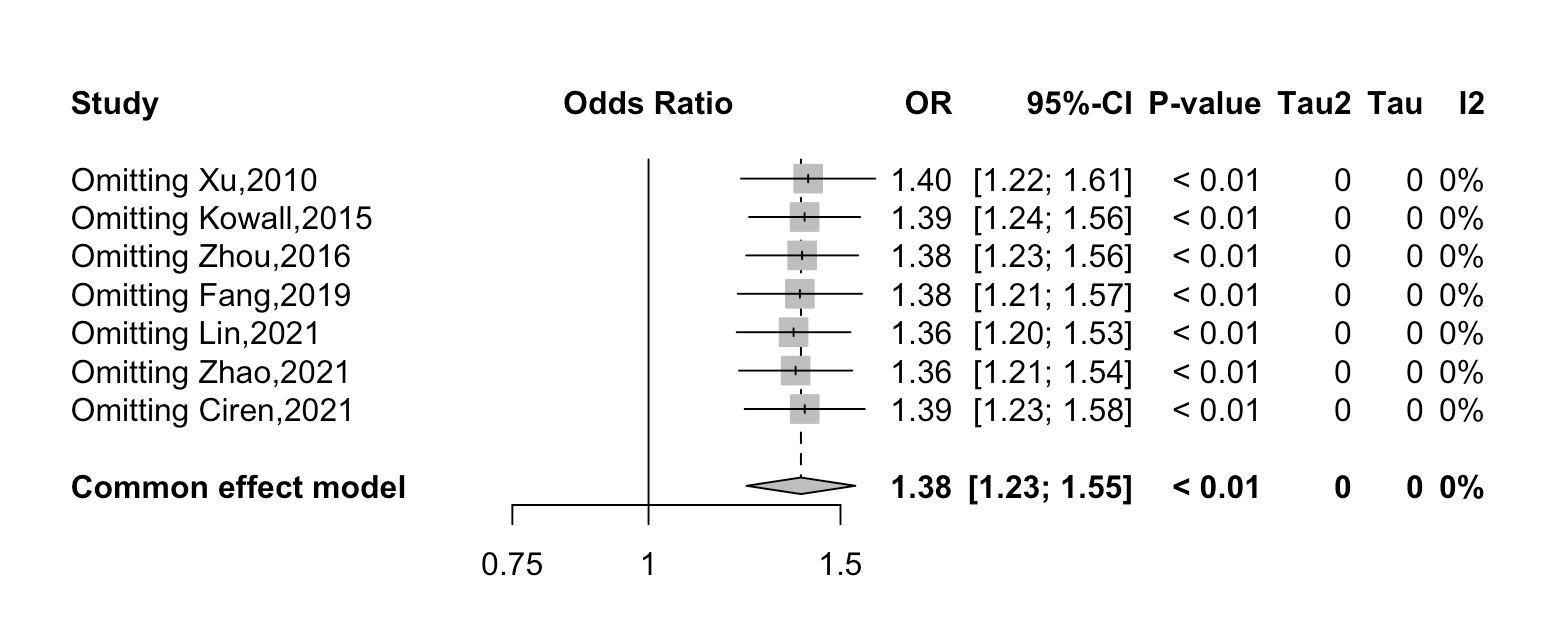


**Supplementary Figure 11.** Sensitivity analysis for the association between napping≥1 h min and risk of T2DM.

## Supplementary Tables

| **Supplementary Table 1.** Search strategy and the results of literature searching | | |
| --- | --- | --- |
| **Databases** | **Search strategies** | **Records** |
| **PubMed** | (nap[MeSH Terms] OR napping[MeSH Terms] OR nap duration[MeSH Terms] OR siesta[MeSH Terms] OR daytime napping[MeSH Terms] OR afternoon napping[MeSH Terms] OR midday napping[MeSH Terms] OR day napping[MeSH Terms] OR habitual afternoon napping[MeSH Terms] OR nap[Title/Abstract] OR napping[Title/Abstract] OR nap duration[Title/Abstract] OR siesta[Title/Abstract] OR daytime napping[Title/Abstract] OR afternoon napping[Title/Abstract] OR midday napping[Title/Abstract] OR day napping[Title/Abstract] OR habitual afternoon napping[Title/Abstract]) AND (diabetes[MeSH Terms] OR diabetes mellitus[MeSH Terms] OR type 2 diabetes mellitus[MeSH Terms] OR type 2 diabetes[MeSH Terms] OR diabetes[Title/Abstract] OR diabetes mellitus[Title/Abstract] OR type 2 diabetes mellitus[Title/Abstract] OR type 2 diabetes[Title/Abstract]) | 192 |
| **Embase** | ('nap':ab,ti OR 'napping':ab,ti OR 'nap duration':ab,ti OR 'siesta':ab,ti OR 'daytime napping':ab,ti OR 'afternoon napping':ab,ti OR 'midday napping':ab,ti OR 'day napping':ab,ti OR 'habitual afternoon napping':ab,ti) AND ('diabetes':ab,ti OR 'diabetes mellitus':ab,ti OR 'dm':ab,ti OR 'type 2 diabetes mellitus':ab,ti OR 'type 2 diabetes':ab,ti OR 't2d':ab,ti OR 't2dm':ab,ti) AND 'article'/it | 165 |
| **Web of science** | (TS=(nap or napping or nap duration or siesta or daytime napping or afternoon napping or midday napping or day napping or habitual afternoon napping) AND TS=(diabetes or diabetes mellitus or DM or type 2 diabetes mellitus or type 2 diabetes or T2D or T2DM)) AND DT=(Article) | 202 |

| **Supplementary Table 2.**  Characteristics of the studies included in the meta-analysis | | | | | | | | |
| --- | --- | --- | --- | --- | --- | --- | --- | --- |
| Author, year | Country | Study | Sample population (N) | T2DM events (%) | Age, years (range) | Females (%) | Napping prevalence (%) | Napping classification |
| Ciren, 2021 | China | cross-sectional study | 2,902 | 21.6 | 45-79 | 57.2 | 27.8 | 0, 1-59 and ≥60 min |
| Lin, 2021 | China | cohort study | 2,620 | 13.7 | ≥60 | 49 | 52.9 | 0, ≤1 and >1 h |
| Zhao, 2021 | China | cross-sectional study | 3,236 | 7.8 | 52.5 | 62.5 | 60.7 | 0, ≤0.5, 0.5-1 and >1 h |
| Fang, 2019 | China | cross-sectional study | 6,940 | 9.7 | 61 | 100 | 53.4 | 0, 1-60 and >60 min |
| Liu, 2018 | China | cross-sectional study | 19,257 | 9.65 | 55.93 | 63.6 | 70 | 0, 1-30, 31-60, 61-90 and ≥91 min |
| Yin, 2018 | China | cross-sectional study | 12,277 | 6.7 | 59.2 | 51.78 | 53.39 | 0, <30, 31-90 and >90 min |
| Liu, 2017 | China | cross-sectional study | 12,663 | 10.31 | 35-74 | 65.53 | 68.23 | 0, 1-30, 31-60, 61-90 and ≥91 min |
| Kowall, 2016 | Germany | cohort study | 2,962 | 8.6 | 45-75 | 45.71 | 49.43 | 0, <30, 30-60 and ≥60min |
| Han, 2016 | China | cohort study | 16,399 | 6.84 | 62.5 | 56.81 | 66.77 | 0, 1-30, 31-60, 61-90 and >90 min |
| Zhou, 2016 | China | cross-sectional study | 13,469 | 1.1 | 55.2 | 50.8 | 51.7 | 0, 1-59, 60 and >60 min |
| Fang, 2013 | China | cross-sectional study | 27,009 | 12.77 | 63.6 | 55.36 | 68.55 | 0, <30, 30-60, 60-90 and ≥90 min |
| Xu, 2010 | USA | cohort study | 174,542 | 5.81 | 50-71 | 43.15 | 45.89 | 0, <1 and ≥1 h |
| Liu, 2022 | China | cohort study | 11,539 | 6.01 | 61 | 64.96 | 42.13 | 0, 1-29, 30-59, 60-89, and ≥90 min |
| Ye, 2019 | China | cross-sectional study | 33,850 | 22.78 | 58.1 | 66.92 | 33.66 | 0, 0-1, 1-1.5, and >1.5 h |

| **Supplementary Table 3.** Summary *OR* estimates (95% *CI*) for including studies of the association between napping duration and risk of T2DM. | | | | | |
| --- | --- | --- | --- | --- | --- |
| Type | No. of studies | OR (95%CI) | *I^2^* (%) | *P-_Heter_* | *P-_Egger_* |
| <30 min | 7 | 1.01 (0.92, 1.10) | 0 | 0.5501 | 0.2478 |
| 30-60 min | 8 | 1.09 (1.02, 1.16) | 0 | 0.8703 | 0.6276 |
| 60-90 min | 5 | 1.07 (1.00, 1.15) | 0 | 0.5922 | 0.3334 |
| >90 min | 6 | 1.20 (1.13, 1.28) | 26 | 0.2417 | 0.0049 |
| <1 h | 6 | 1.16 (1.12, 1.22) | 0 | 0.7605 | 0.6370 |
| ≥1 h | 8 | 1.38 (1.23, 1.55) | 0 | 0.9803 | 0.7826 |

| **Supplementary Table 4.** Assess quality using the Agency for Healthcare Research and Quality | | | | | | | | | | | | |
| --- | --- | --- | --- | --- | --- | --- | --- | --- | --- | --- | --- | --- |
| Author, Year | Item 1 | Item 2 | Item 3 | Item 4 | Item 5 | Item 6 | Item 7 | Item 8 | Item 9 | Item 10 | Item 11 | Total |
| Ciren, 2021 | yes | yes | yes | yes | no | yes | yes | yes | unclear | yes | no | 8 |
| Fang, 2019 | yes | yes | yes | yes | no | no | yes | yes | unclear | yes | no | 7 |
| Fang, 2013 | yes | no | yes | yes | no | no | no | yes | no | yes | no | 5 |
| Liu, 2018 | yes | yes | no | yes | no | yes | yes | yes | no | yes | no | 7 |
| Liu, 2017 | yes | yes | no | yes | no | yes | yes | yes | no | yes | no | 7 |
| Yin, 2018 | yes | yes | yes | yes | no | no | yes | yes | no | yes | no | 7 |
| Zhao, 2021 | yes | yes | yes | yes | no | yes | yes | yes | no | yes | no | 8 |
| Zhou, 2016 | yes | yes | yes | yes | no | yes | yes | yes | no | yes | no | 8 |
| Ye, 2019 | yes | yes | no | yes | no | yes | yes | yes | no | yes | no | 7 |

| **Supplemental Table 5.** Assess quality using the Newcastle-Ottawa quality assessment scale | | | | | | | | | |
| --- | --- | --- | --- | --- | --- | --- | --- | --- | --- |
|  | Study design | | | | Comparability | Outcome | | |  |
| Author, Year | Item 1 | Item 2 | Item 3 | Item 4 | Item 5 | Item 6 | Item 7 | Item 8 | Total |
| Kowall, 2016 | ☆ | ☆ |  | ☆ | ☆☆ |  | ☆ | ☆ | 7 |
| Lin, 2021 | ☆ | ☆ |  | ☆ | ☆☆ | ☆ | ☆ |  | 7 |
| Xu, 2010 | ☆ | ☆ |  | ☆ | ☆☆ |  | ☆ | ☆ | 7 |
| Han, 2016 | ☆ | ☆ |  | ☆ | ☆☆ |  | ☆ |  | 6 |
| Liu, 2022 | ☆ | ☆ |  | ☆ | ☆ |  | ☆ | ☆ | 6 |

**Supplementary Table 6.** PRISMA 2020 Main Checklist

| **Topic** | **No.** | **Item** | **Location where item is reported** |
| --- | --- | --- | --- |
| **TITLE** |  |  |  |
| **Title** | 1 | Identify the report as a systematic review. | NA. |
| **ABSTRACT** |  |  |  |
| **Abstract** | 2 | See the PRISMA 2020 for Abstracts checklist | NA. |
| **INTRODUCTION** |  |  |  |
| **Rationale** | 3 | Describe the rationale for the review in the context of existing knowledge. | NA. |
| **Objectives** | 4 | Provide an explicit statement of the objective(s) or question(s) the review addresses. | NA. |
| **METHODS** |  |  |  |
| **Eligibility criteria** | 5 | Specify the inclusion and exclusion criteria for the review and how studies were grouped for the syntheses. | Page S1 (1.2 study selection) |
| **Information sources** | 6 | Specify all databases, registers, websites, organisations, reference lists and other sources searched or consulted to identify studies. Specify the date when each source was last searched or consulted. | Page S1 (1.1 Literature search) |
| **Search strategy** | 7 | Present the full search strategies for all databases, registers and websites, including any filters and limits used. | Page S9 (Table S1) |
| **Selection process** | 8 | Specify the methods used to decide whether a study met the inclusion criteria of the review, including how many reviewers screened each record and each report retrieved, whether they worked independently, and if applicable, details of automation tools used in the process. | Page S1 (1.2 study selection) |
| **Data collection process** | 9 | Specify the methods used to collect data from reports, including how many reviewers collected data from each report, whether they worked independently, any processes for obtaining or confirming data from study investigators, and if applicable, details of automation tools used in the process. | Page S1 (1.3 Data extraction and quality) |
| **Data items** | 10a | List and define all outcomes for which data were sought. Specify whether all results that were compatible with each outcome domain in each study were sought (e.g. for all measures, time points, analyses), and if not, the methods used to decide which results to collect. | Page S1 (1.3 Data extraction and quality) |
|  | 10b | List and define all other variables for which data were sought (e.g. participant and intervention characteristics, funding sources). Describe any assumptions made about any missing or unclear information. | Page S1 (1.3 Data extraction and quality) |
| **Study risk of bias assessment** | 11 | Specify the methods used to assess risk of bias in the included studies, including details of the tool(s) used, how many reviewers assessed each study and whether they worked independently, and if applicable, details of automation tools used in the process. | Page S1 (1.3 Data extraction and  quality) |
| **Effect measures** | 12 | Specify for each outcome the effect measure(s) (e.g. risk ratio, mean difference) used in the synthesis or presentation of results. | Page S1-2 (1.4 Data synthesis and analysis) |
| **Synthesis methods** | 13a | Describe the processes used to decide which studies were eligible for each synthesis (e.g. tabulating the study intervention characteristics and comparing against the planned groups for each synthesis (item 5)). | Page S1-2 (1.4 Data synthesis and analysis) |
|  | 13b | Describe any methods required to prepare the data for presentation or synthesis, such as handling of missing summary statistics, or data conversions. | Page S1-2 (1.4 Data synthesis and analysis) |
|  | 13c | Describe any methods used to tabulate or visually display results of individual studies and syntheses. | Page S1-2 (1.4 Data synthesis and analysis) |
|  | 13d | Describe any methods used to synthesize results and provide a rationale for the choice(s). If meta-analysis was performed, describe the model(s), method(s) to identify the presence and extent of statistical heterogeneity, and software package(s) used. | Page S1-2 (1.4 Data synthesis and analysis) |
|  | 13e | Describe any methods used to explore possible causes of heterogeneity among study results (e.g. subgroup analysis, meta-regression). | Page S1-2 (1.4 Data synthesis and analysis) |
|  | 13f | Describe any sensitivity analyses conducted to assess robustness of the synthesized results. | Page S1-2 (1.4 Data synthesis and analysis) |
| **Reporting bias assessment** | 14 | Describe any methods used to assess risk of bias due to missing results in a synthesis (arising from reporting biases). | Page S1-2 (1.4 Data synthesis and analysis) |
| **Certainty assessment** | 15 | Describe any methods used to assess certainty (or confidence) in the body of evidence for an outcome. | NA. |
| **RESULTS** |  |  |  |
| **Study selection** | 16a | Describe the results of the search and selection process, from the number of records identified in the search to the number of studies included in the review, ideally using a flow diagram. | Page S2 (Figure S1) |
|  | 16b | Cite studies that might appear to meet the inclusion criteria, but which were excluded, and explain why they were excluded. | Page S2 (Figure S1) |
| **Study characteristics** | 17 | Cite each included study and present its characteristics. | Page S10-11 (Table S2) |
| **Risk of bias in studies** | 18 | Present assessments of risk of bias for each included study. | Page S 12 (Table S4-5) |
| **Results of individual studies** | 19 | For all outcomes, present, for each study: (a) summary statistics for each group (where appropriate) and (b) an effect estimate and its precision (e.g. confidence/credible interval), ideally using structured tables or plots. | Page S3-6 (Figure S2-5) |
| **Results of syntheses** | 20a | For each synthesis, briefly summarise the characteristics and risk of bias among contributing studies.6 | Page S3-6 (Figure S2-5) |
|  | 20b | Present results of all statistical syntheses conducted. If meta-analysis was done, present for each the summary estimate and its precision (e.g. confidence/credible interval) and measures of statistical heterogeneity. If comparing groups, describe the direction of the effect. | Page S3-6 (Figure S2-5) |
|  | 20c | Present results of all investigations of possible causes of heterogeneity among study results. | Page S3-5 (Figure S2-5) |
|  | 20d | Present results of all sensitivity analyses conducted to assess the robustness of the synthesized results. | Page S7-9 (Figure S6-11) |
| **Reporting biases** | 21 | Present assessments of risk of bias due to missing results (arising from reporting biases) for each synthesis assessed. | Page S11 (Table S3) |
| **Certainty of evidence** | 22 | Present assessments of certainty (or confidence) in the body of evidence for each outcome assessed. | NA. |
| **DISCUSSION** |  |  |  |
| **Discussion** | 23a | Provide a general interpretation of the results in the context of other evidence. | NA. |
|  | 23b | Discuss any limitations of the evidence included in the review. | NA. |
|  | 23c | Discuss any limitations of the review processes used. | NA. |
|  | 23d | Discuss implications of the results for practice, policy, and future research. | NA. |
| **OTHER INFORMATION** |  |  |  |
| **Registration and protocol** | 24a | Provide registration information for the review, including register name and registration number, or state that the review was not registered. | NA. |
|  | 24b | Indicate where the review protocol can be accessed, or state that a protocol was not prepared. | NA. |
|  | 24c | Describe and explain any amendments to information provided at registration or in the protocol. | NA. |
| **Support** | 25 | Describe sources of financial or non-financial support for the review, and the role of the funders or sponsors in the review. | Page 12 (Funding source) |
| **Competing interests** | 26 | Declare any competing interests of review authors. | Page 12 (Declaration of Competing Interest) |
| **Availability of data, code and other materials** | 27 | Report which of the following are publicly available and where they can be found: template data collection forms; data extracted from included studies; data used for all analyses; analytic code; any other materials used in the review. | Supplementary Materials |
